# Direct interaction between fd phage pilot protein pIII and the TolQ–TolR proton-dependent motor provides new insights into the import of filamentous phages

**DOI:** 10.1016/j.jbc.2023.105048

**Published:** 2023-07-13

**Authors:** Callypso Pellegri, Ambre Moreau, Denis Duché, Laetitia Houot

**Affiliations:** Laboratoire d'Ingénierie des Systèmes Macromoléculaires, UMR7255, Institut de Microbiologie de la Méditerranée, Aix-Marseille Univ – CNRS, Marseille Cedex, France

**Keywords:** filamentous bacteriophage, Tol–Pal system, infection, protein–protein interaction, proton motive force, virus entry

## Abstract

Filamentous phages are one of the simplest examples of viruses with a protein capsid that protects a circular single-stranded DNA genome. The infection is very specific, nonlytic, and can strongly affect the physiology or provide new pathogenic factors to its bacterial host. The infection process is proposed to rely on a pore-forming mechanism similar to that of certain nonenveloped eukaryotic viruses. The Ff coliphages (including M13, fd, and f1) have been intensively studied and were used to establish the sequence of events taking place for efficient crossing of the host envelope structure. However, the mechanism involved in the penetration of the cell inner membrane is not well understood. Here, we identify new host players involved in the phage translocation mechanism. Interaction studies by a combination of *in vivo* biochemical methods demonstrate that the adhesion protein pIII located at the tip of the phage binds to TolQ and TolR, two proteins that form a conserved proton-dependent molecular motor in the inner membrane of the host cell. Moreover, *in vivo* cysteine cross-linking studies reveal that the interactions between the pIII and TolQ or TolR occur between their transmembrane helix domains and may be responding to the proton motive force status of the cell. These results allow us to propose a model for the late stage of filamentous phage translocation mediated by multiple interactions with each individual component of the host TolQRA complex.

Bacteriophages disseminate from host to host, shaping bacterial communities in all environments on earth. Most of these viruses are equipped with molecular machineries and enzymatic domains to specifically puncture their host’s envelope and inject their genome into the cell cytoplasm. Successful infection converts the host to a factory dedicated to the assembly of the phage progeny that can be finally released upon lysis of the host. Filamentous phages, however, have a unique life cycle, as they behave like symbionts, establishing a chronic infection and extruding progeny virions in the environment by secretion without killing the host. These viruses infect gram-negative bacteria by parasitizing the cell envelope structures to target their host and to cross the outer membrane (OM) and the periplasm. Final uncapping of the phage particle and penetration into the inner membrane (IM) is required to complete the viral infection. However, the molecular strategy for viral genome transport from the particle to the cytoplasm is still speculative (1).

Notorious filamentous phages have been described for their ability to increase the virulence potential of their bacterial host, including the sepsis and meningitis agent *Escherichia coli* O18:K1:H7, the plague *bacillus Yersinia pestis*, the meningococcal agent *Neisseria meningitidis*, and the cholera agent *Vibrio cholerae* (2, 3, 4, 5), to participate in biofilm building and resistance to environmental stresses and to improve escape from the immune system (*e.g.*, *Pseudomonas aeruginosa*) (6). Recently, filamentous phage secretion has been proven to delay healing of infected wounds, highlighting a potential interest in vaccines targeting phage particles to fight infections (7). Their potential as biocontrol agent was investigated for the crop pathogen *Erwinia* (8). Despite the intensive use of the canonical coliphage M13 (or fd) in the fields of molecular biology and phage display (9, 10), the molecular mechanisms of infection responsible for the spreading of these viruses among bacterial populations remain unclear.

The canonical coliphage particle fd is composed of 2700 copies of the major coat protein pVIII that covers the genomic circular ssDNA. Five units of the minor coat proteins cap both ends of the virus, pVII and pIX on one side, pIII and pVI on the other side (designed as the phage “head”) (Fig. 1). In the producing cell, all the capsid proteins are synthesized and accumulated in the IM *via* transmembrane helices (TMHs) before being assembled into the new virions (1). A macrocomplex composed of the phage-encoded proteins pI, pIV, and pXI spans the whole envelope (11) and allows assembly and secretion of the progeny virion in the environment, starting with the pVII–pIX complex, followed by an helical assembly of pVIII on the ssDNA. A precomplex of pIII–pVI finalizes the structure and allows its detachment from the envelope. Recently, Conners *et al.* (12) obtained the first cryo-EM structure for f1 nanophages, providing new insights into the organization of both major and minor capsid proteins in the viral particle.

**Figure 1:**
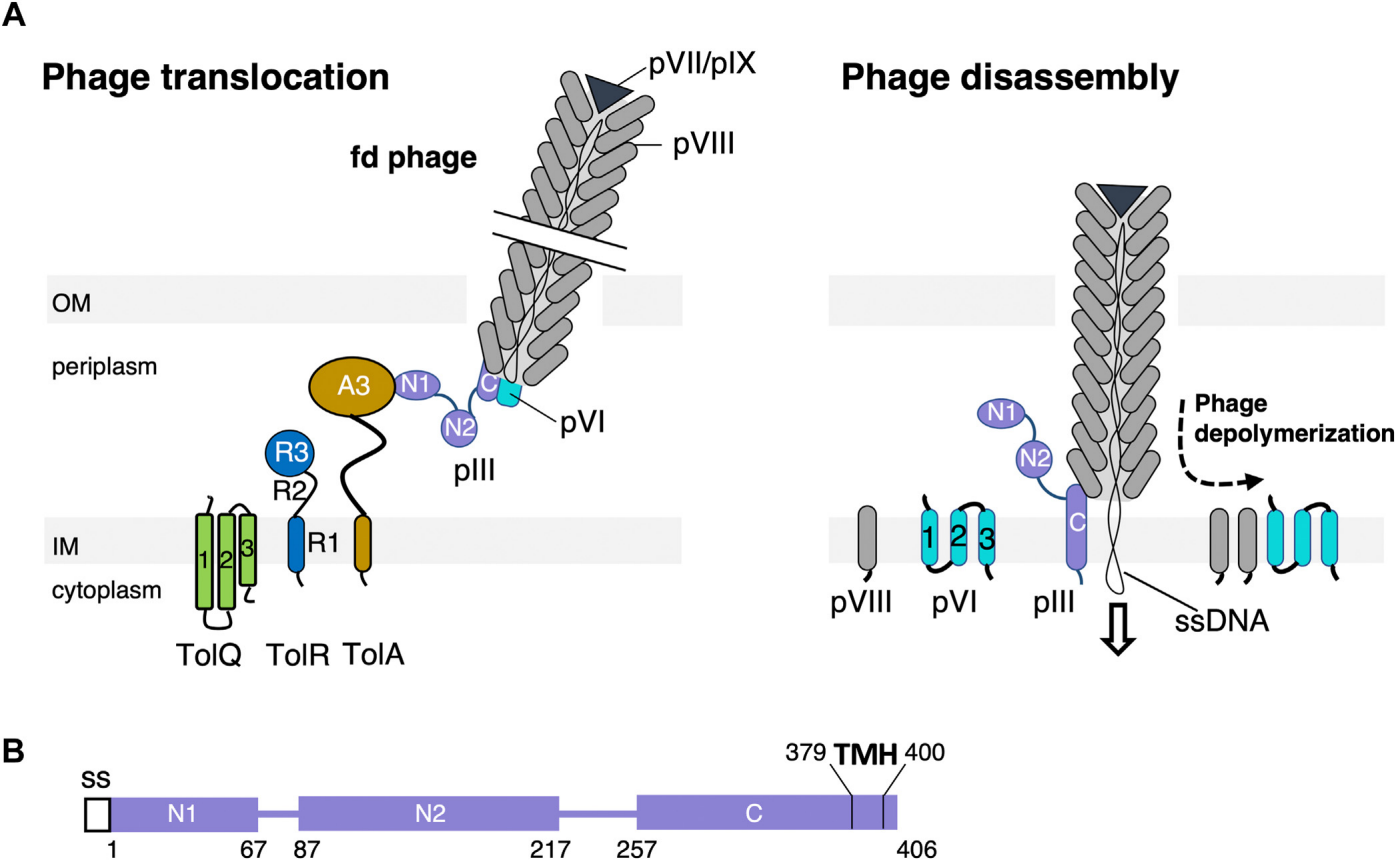
**Schematic representation of the phage import process.***A*, the TolA-dependent translocation step (*left*) and the final phage disassembly in the IM (*right*) are presented, highlighting the localizations and suggested topologies of the proteins of interest. The different protein domains are presented according to previously published literature. The phage head is composed of five copies of pIII and pVI, only one of each being shown in the figure. The F-pilus apparatus, involved in the initial phage reception step, and the peptidoglycan layer are omitted for clarity. *B*, schematic representation of the three domains alongside the residue numbering for the mature protein. IM, inner membrane; OM, outer membrane; SS, signal sequence; TMH, transmembrane helix.

The model for filamentous phage infection involves a depolymerization process that somehow mirrors the secretion mechanism. Two sequential bacterial receptors have been identified: the conjugative F-pilus that expands from the cell surface and TolA and the pivot protein of the Tol complex in the IM (Fig. 1*A*). The orchestrator of the phage import is the pIII protein located with pVI at the tip of the phage. pIII is organized into three domains N1, N2, and C, which are separated by glycine-/serine-rich linkers (Fig. 1*B*). Structures of pIII-N1 and pIII-N2 soluble domains have been determined (13, 14) and are not visible in the full viral structure, most likely because of the flexibility of the linkers (12). The current infection model involves a three-step mechanism. In the reception step, the phage binds the tip of the conjugative F-pilus through the pIII-N2 domain (15). Pilus retraction has been proposed to pull the phage close to the envelope surface and possibly through the OM *via* the pilus secretin. This step facilitates phage infection but is not strictly required, as the F^−^ cells can still be infected with low efficiency (16). During the translocation step, phage crossing of the periplasmic space relies on proteins of the Tol system. Indeed, the three IM anchored proteins, TolQ, TolR, and TolA, form a complex required for infection (17, 18). A direct interaction between the TolA globular domain (TolA3) and the phage pIII-N1 domain has been characterized by structural approaches (13, 19). The role of TolQ or TolR in the infection process is unclear. The final phage genome injection step requires opening of the phage head and insertion of the pIII C-terminal hydrophobic segment in the IM (Fig. 1*A*). pIII-C may assemble as a pentamer that possibly forms a pore for the phage ssDNA genome to cross the IM. Indeed purified pIII proteins can form pores in artificial lipid bilayers (20). Deletion of a 28-residue-long segment in pIII-C (residues 286–314) strongly reduces the infectivity of the phage, whereas deletion of a longer segment (residues 286–324) renders the phage noninfective (21, 22). It has been suggested that pVI may participate in the formation of the pore (12), although this hypothesis remains to be proven experimentally. In the final step, sequential disassembly of the pVIII capsid proteins in the lipid bilayer of the host is proposed to be concomitant to viral genome transfer into the cytoplasm and to its conversion in a dsDNA replicative form (23).

The phage particle is a very robust structure and cannot be destabilized by exposure to artificial membranes or to cell membrane fractions obtained by mechanical disruption (23). Moreover, based on the predicted structure of the intact phage particle, pIII and pVI would need to undergo significant conformational changes to dive into the host membrane (12). Thus, we investigated the hypothesis that the critical step of the virion head “opening” is occurring through structural modifications driven by sequential protein–protein interactions with the host proteins. TolA, TolQ, and TolR proteins form the IM complex of the Tol–Pal system, a proton motive force (PMF)-dependent molecular motor conserved in gram-negative bacteria (Fig. 1*A*). The TolQ–TolR motor is evolutionary related to the ExbB–ExbD motor of the Ton system, which is involved in the active transport of iron siderophores and cobalamin (24). TolA and TolR are both anchored to the IM *via* one N-terminal TMH, whereas TolQ is embedded *via* three TMHs, named Q1, Q2, and Q3. Based on biochemical analyses (25) and by homology with the ExbB–ExbD motor (26, 27), TolQ may form a pentameric structure surrounding a dimer of TolR proteins, with Q2 and Q3 interacting with TolR TMHs (R1 TMH). TolR dimerizes through R1 TMH into the IM and through R2 and R3 domains in the periplasm (28, 29). It has been suggested that the assembly of the TolQ–TolR motor induces conformational changes and exposure of a peptidoglycan-binding domain in TolR3 (30). In the IM, the dynamic interactions between Q2, Q3, and R1 TMHs define two proton channels that might sequentially operate. Biochemical studies, structural data, and modelization suggest that TolA TMH (TolA1) interacts with TolQ1 at the periphery of the motor (31, 32, 33). The flux of protons is converted into mechanical movements transduced to the TolA protein, which interacts with the OM lipoprotein Pal and with the periplasmic protein TolB (34, 35, 36). In *E. coli*, mutants of the Tol–Pal system display numerous defects collectively called *tol* phenotypes, such as a chaining morphology in low osmotic medium, hypersensitivity to detergents, resistance to Tol-dependent toxins called colicins (37), and increased resistance to chromate (38). Thorough analysis of these pleiotropic phenotypes over the past 50 years demonstrated that the Tol–Pal complex is crucial in envelope integrity maintenance, OM lipid homeostasis, and in the late stage of cell division (39, 40, 41, 42, 43).

We recently showed that phage uptake in the host required an assembled TolA–TolQ–TolR complex. Strains producing TolQ point mutants defective for PMF utilization by the Tol system show *tol* phenotypes but remain efficient for filamentous phage import (18). We also demonstrated that the ExbB–ExbD proteins can crossoperate with TolA to drive phage import in the absence of the TolQ–TolR proteins, however without restoring the functionality of the Tol system.

In this study, we provide molecular details on the interactions occurring between the phage head protein pIII and the host IM complex TolQ–TolR–TolA during infection. Bacterial two-hybrid (BACTH), coimmunoprecipitation (co-IP) experiments, and a partial proteolysis assay show that the phage minor capsid protein pIII interacts with TolQ and TolR. Using shorter variants of pIII, we demonstrate that the pIII C-terminal domain is sufficient to interact with TolQ and TolR. Finally, *in vivo* cysteine cross-linking studies reveal that binding between the phage and the host proteins involves their TMH domains, some of the interactions responding to the PMF status of the cell.

## Results

### Interaction studies of the minor coat proteins constituting the fd phage head

**Figure 2:**
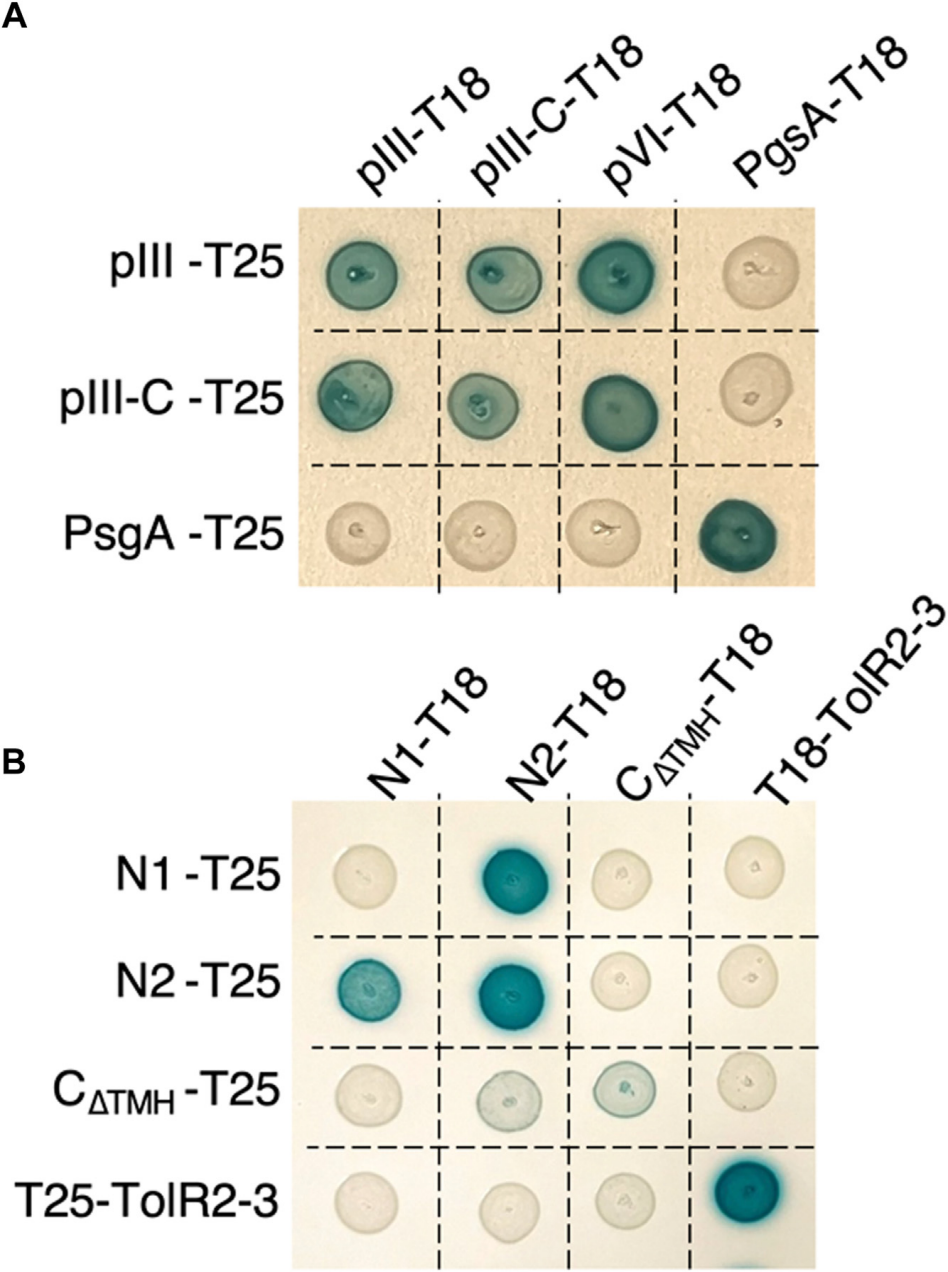
**Interaction studies of the phage minor coat proteins pIII and pVI.** Bacterial two-hybrid assays were performed in the BTH101 (*A*) or Oxi-Blue (*B*) reporter strains producing the indicated proteins or domains (pIII-C, residues 256–406; N1, residues 1–71; N2, residues 82–222; C_ΔTMH_, residues 256–378 of the pIII protein; TolR2–3, residues 45–143 of TolR) fused to the T18 or T25 domain of the *Bordetella* adenylate cyclase. Cells were spotted on plates supplemented with IPTG and X-Gal. Interaction between the two fusion proteins is attested by the *blue color*. The PgsA–PgsA (*A*) and TolB–Pal (*B*) interactions serve as positive controls. TMH, transmembrane helix.

The phage relies on its pIII cap proteins to orchestrate targeting and penetration of the host envelope. While pIII-N1 and pIII-N2 bind to TolA3 and to the F-pilus, respectively, it is not known how pIII-C is extracted from the viral particle to insert into the IM. In the intact phage head, the five pIII C-domains are mainly organized in α-helices in close contact with five units of pVI. A pIII-C intrachain disulfide bond between Cys-354 and Cys-371 stabilizes a β-hairpin upstream the hydrophobic terminal α-helix (12). The capsid proteins all have the IM of the host as their final destination. We first aimed to get a better understanding of the interactions occurring between pIII, pVI, pVIII proteins, and the pIII subdomains using a BACTH approach. First, the full-length *g3p*, *g8p*, and *g6p* genes of the fd phage were cloned in frame with the T18 and T25 domains of the *Bordetella pertussis* adenylate cyclase at their C terminus. We predicted that the chimeric constructs would localize in the IM through their hydrophobic segments, with the T18 and T25 domains exposed in the cytoplasm. The assays conducted in the BTH101 strain on reporter plates demonstrated that pIII interacts with itself as well as with pVI (Fig. 2*A*) as expected (44, 45). A pIII-C construct comprising residues 256 to 406, which correspond to pIII deleted of its N1 and N2 domains, was sufficient for both homo-oligomerization and heteromerization with pVI (Fig. 2*A*). Of note, our attempt to include the major coat protein pVIII to this BACTH study failed as the pVIII-T18 and pVIII-T25 fusions displayed unspecific binding to control membrane proteins (data not shown).

The sequence encoding pIII-N1 (residues 1–71 of the mature pIII protein), pIII-N2 (residues 82–222), and pIII-C deleted of its TMH (C_ΔTM_; residues 256–378) were fused in frame with T18 and T25 in the BACTH plasmids. As the N1 and C_ΔTM_ domains comprise two and one disulfide bonds, respectively, the Oxi-Blue strain was used to conduct the two-hybrid assay; this background derived from the Oxi-BTH strain promotes correct folding of disulfide bond–structured proteins in its cytoplasm (46). As previously reported by crystallography (14) and surface plasmon resonance analysis (47), we observed in the BACTH assay that pIII-N1 interacts with pIII-N2 (Fig. 2*B*), attesting to the correct production and folding of the constructs. pIII-N2 but not pIII-N1 was able to oligomerize. Finally, we observed that deletion of the pIII-C TMH strongly impaired the ability of pIII-C_ΔTM_ to oligomerize despite correct production of the proteins (Fig. S1).

### pIII interacts with TolR and TolQ *in vivo*

**Figure 3:**
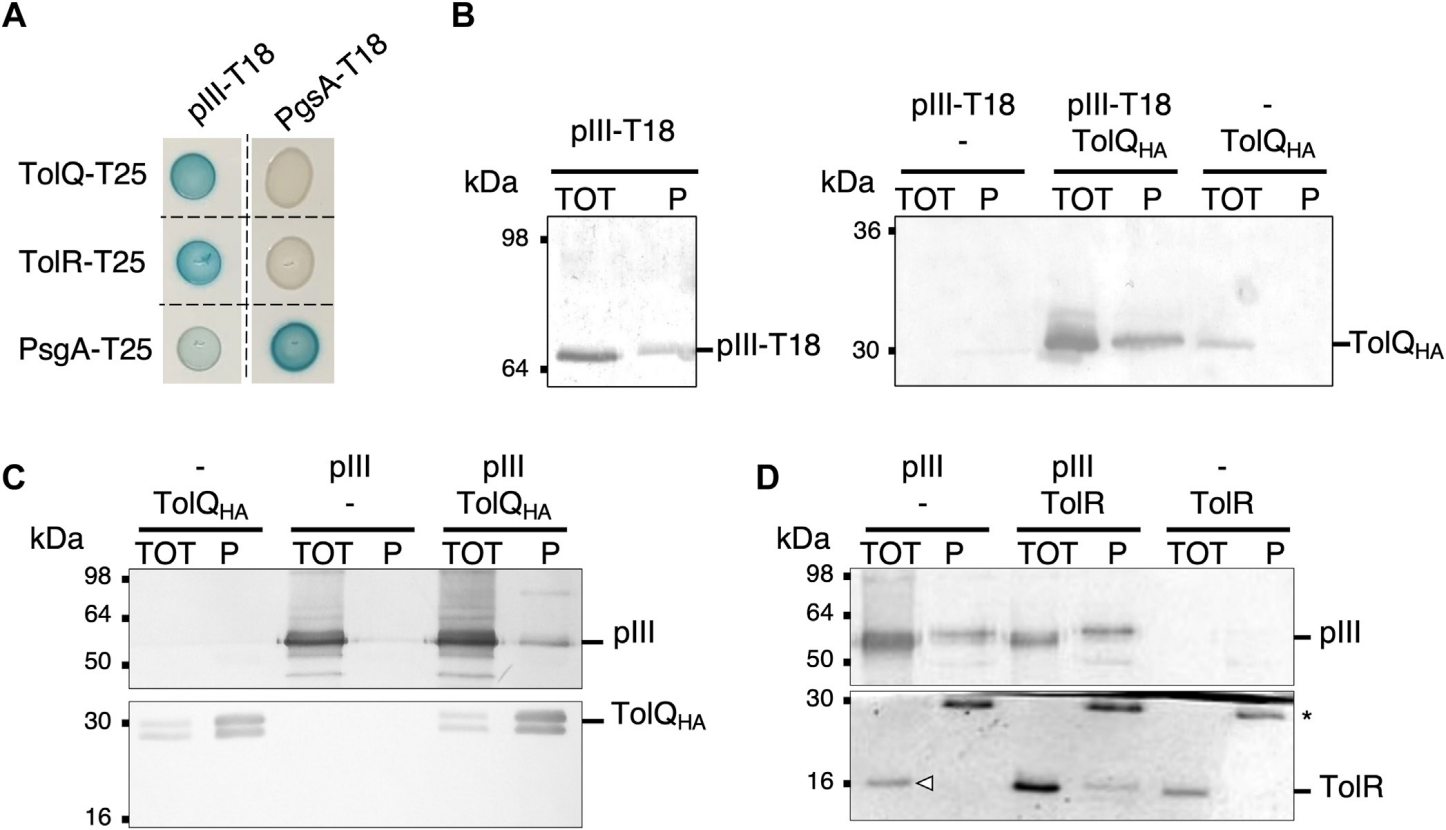
**Interactions between pIII, TolQ, and TolR.***A*, bacterial two-hybrid assay. BTH101 reporter cells producing the indicated proteins fused to the T18 or T25 domain of the *Bordetella* adenylate cyclase were spotted on plates supplemented with IPTG and X-Gal. Interaction between the indicated fusion proteins is attested by the *blue color* signal. The PgsA–PgsA interaction serves as positive controls. *B*, copurification assay. Triton-solubilized extracts of *Escherichia coli* W3110 WT cells producing pIII fused to the adenylate cyclase T18 domain and hemagglutinin (HA)-tagged TolQ were subjected to immobilization on calmodulin beads. *Left panel*, immunodetection of pIII-T18 purification using anti-pIII antibodies. *Right panel*, immunodetection of TolQ copurification using anti-HA antibodies. *C* and *D*, coimmunoprecipitation assays. Triton-solubilized extracts of *E. coli* W3110 WT cells producing the indicated proteins were subjected to immunoprecipitation with anti-HA (*C*) or anti-pIII (*D*) coupled beads. For *B*–*D*, the input (total soluble material, TOT) and the immunoprecipitated material (P) were loaded on a 13.5%-acrylamide SDS-PAGE and immunodetected with anti-HA, anti-TolR, or anti-pIII antibodies. The molecular weight markers are indicated on the *left*. A *white triangle* indicates the signal from the endogenous *tolR* locus. The *star* mark (∗) indicates a protein retained by the beads and nonspecifically detected by the polyclonal anti-TolR antibodies.

Phage infection requires an assembled TolA–TolQ–TolR complex in the IM (18) although pIII-N1–TolA3 is the only direct interaction characterized in the literature (13, 17, 47). We investigated if the TolR and TolQ proteins could also act as receptors of the phage. Using the BACTH assay, we observed that pIII was able to interact with both TolR and TolQ (Fig. 3*A*). The interactions were confirmed by copurification and co-IP. First, plasmids encoding TolQ with a hemagglutinin (HA) tag and pIII fused to the T18 domain were introduced by transformation into an *E. coli* W3110 strain. The membrane proteins were solubilized with a Triton treatment. We purified pIII-T18 on calmodulin beads and observed by immunodetection that TolQ_HA_ specifically copurified with pIII-T18 (Fig. 3*B*). Reciprocally, we showed that the native pIII protein specifically coimmunoprecipitated with TolQ_HA_ using an anti-HA resin (Fig. 3*C*). Finally, TolR overproduced from a vector coimmunoprecipitated with pIII using sepharose beads customized with anti-pIII antibodies (Fig. 3*D*). Of note, endogenous TolR production was not sufficient to visualize the TolR–pIII co-IP signal. It has been previously shown that the homologous ExbB–ExbD motor can replace TolQ–TolR for phage uptake (18). In line with these data, we found that pIII is able to interact with ExbB in a BACTH assay (Fig. S2*A*). Altogether, our data demonstrate direct interactions between the phage pIII protein and both TolQ and TolR proteins that form the Tol motor in the IM of the host. Of note, interactions between the phage protein pVI and TolQ or TolR could not be tested, as pVI stability is dependent on pIII (45). Indeed, pVI-T18 could only be immunodetected when coproduced with pIII in our assays.

### pIII protects the C-terminal domain of TolQ in a proteolysis accessibility assay

**Figure 4:**
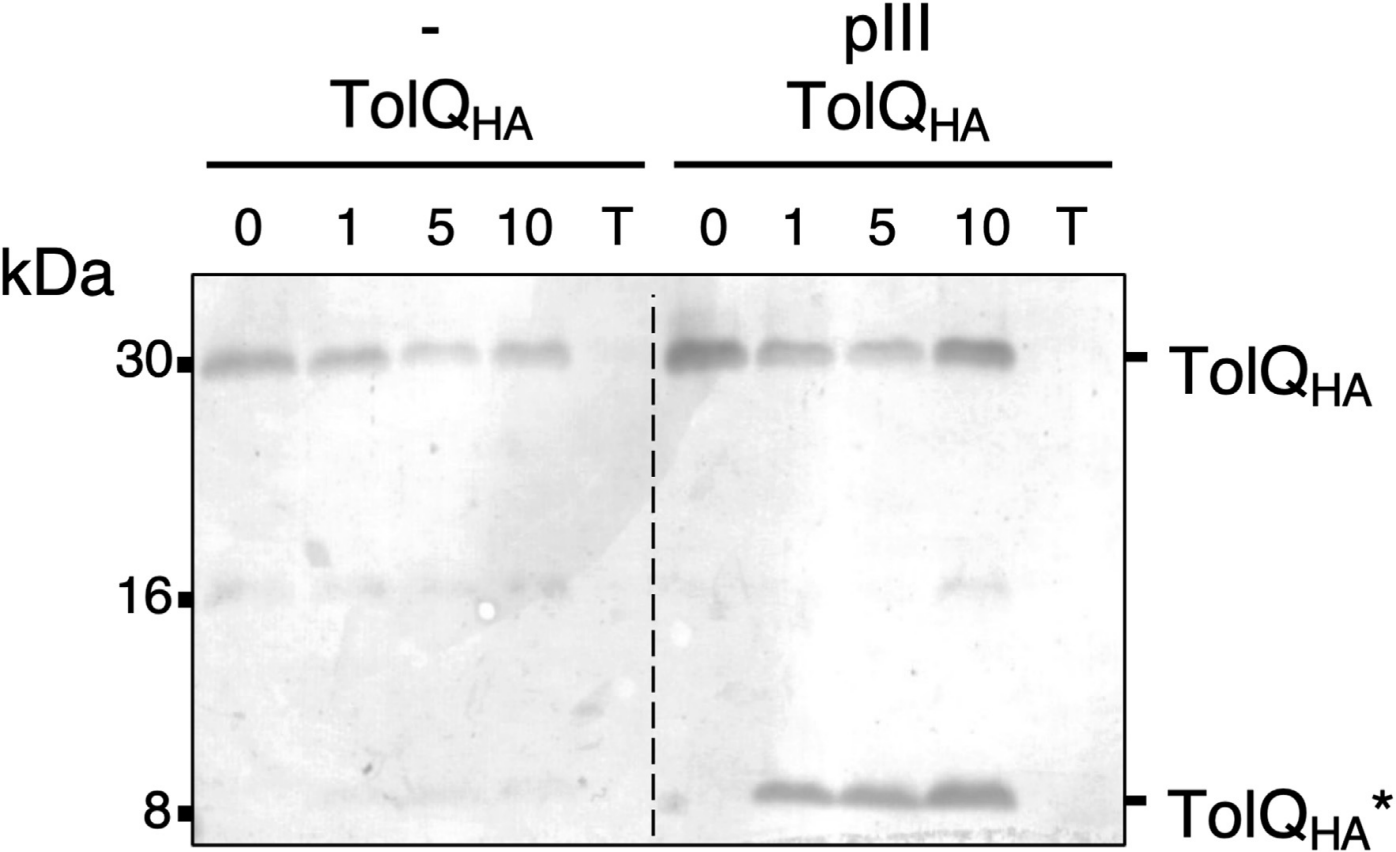
**TolQ**_**HA**_**protease accessibility assay.** TolQ_HA_ was produced in WT *Escherichia coli* cells with or without pIII. Spheroplasts were treated with proteinase K for the indicated time (0, 1, 5, and 10 min). A control sample was incubated with Triton and proteinase K for 10 min to solubilize the membrane proteins (T). TolQ_HA_ was analyzed by SDS-PAGE, and immunoblot was performed with anti-hemagglutinin (HA) antibodies. The full-length TolQ_HA_ protein is indicated as well as the main degradation product TolQ_HA_∗. The molecular mass markers (in kilodalton) are indicated on the *left*.

We next investigated how pIII was interacting with its partners in the bacterial membrane by performing a limited proteolytic digestion of whole cells by proteinase K. First, TolQ tagged with a C-terminal HA epitope and pIII full-length proteins were coproduced from compatible expression vectors. Correct production and localization of pIII in the membrane were attested by cell fractionation and immunodetection (Fig. S3*A*). The pattern of TolQ_HA_ digestion by proteinase K in the absence or in the presence of pIII is shown in Figure 4. A protease-resistant TolQ_HA_ fragment (referred to as TolQ_HA_∗) with an apparent size of 9 kDa could only be visualized when coproduced with the pIII protein. As TolQ_HA_∗ is detected with antibodies raised against the C-terminal HA tag, this suggests that pIII protects approximately the last 80 TolQ residues from proteolysis, which correspond to the periplasmic loop and the Q3 TMH of TolQ (Fig. S2*B*). We also performed proteolysis experiments with TolR produced in the presence or in the absence of pIII. The resulting proteinase K digestion profile detected with a polyclonal anti-TolR antibody did not highlight any obvious protected fragment of TolR by the pIII protein. Reciprocally, the pIII proteolysis profile immunodetected with a polyclonal anti-pIII antibody did not demonstrate any specific pattern in the presence of TolQ or TolR (data not shown).

### The C-terminal hydrophobic helix of pIII is required for binding to TolQ and TolR

**Figure 5:**
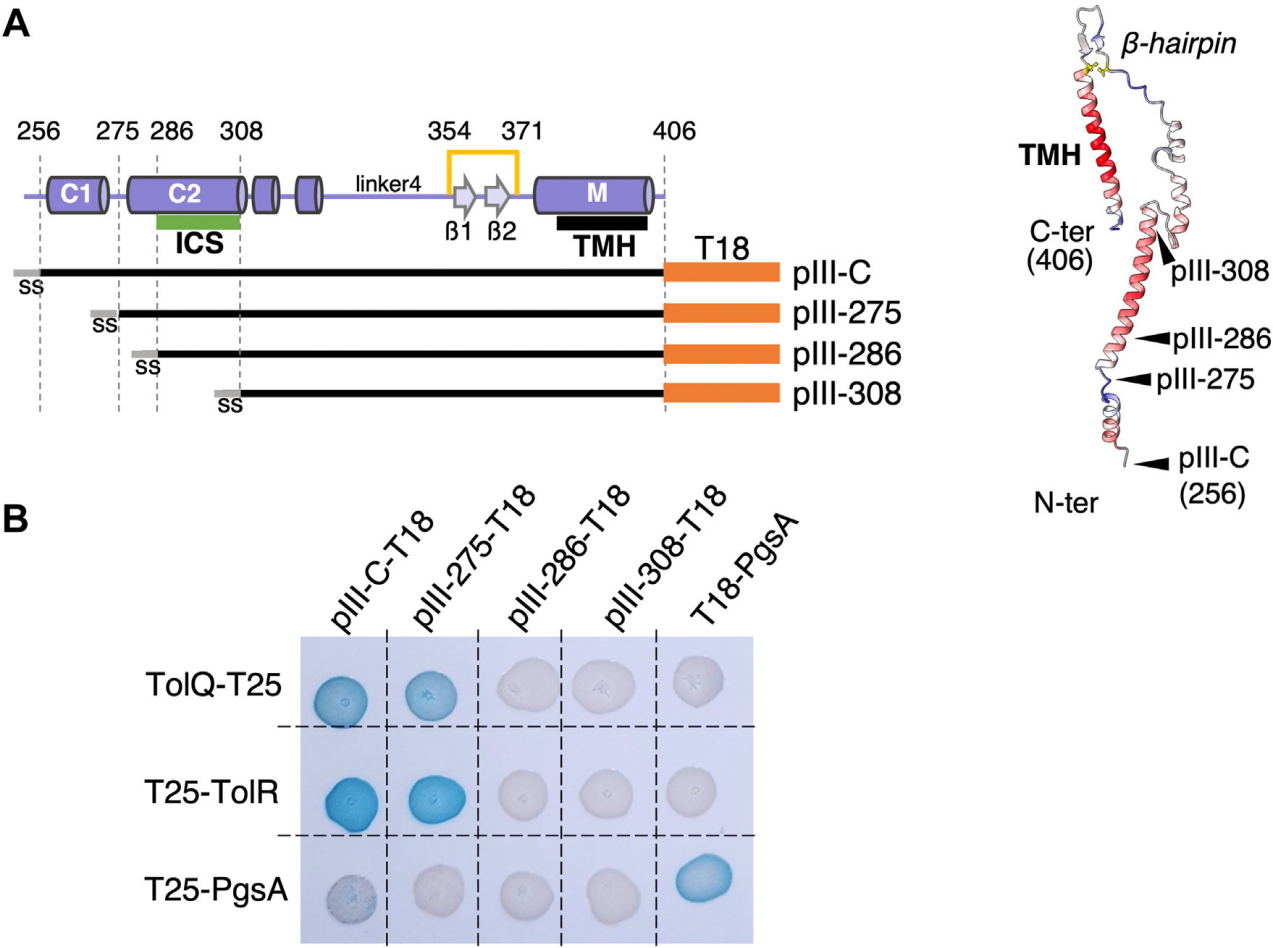
**Protein–protein interactions between Tol and pIII-C variant proteins.***A*, representation of the pIII-C protein sequence and the various truncated constructs tested. Residues are numerated according to the mature protein. *Left panel*, schematic representation with secondary structures indicated as *arrows* (β-strands) or *cylinders* (⍺-helices) and named as previously published (21). The endogenous disulfide bond is schematized by a *yellow line*. SS, the 18-residue signal sequence ensures protein insertion in the IM with periplasmic addressing of the N terminus. *Right panel*, mapping of the positions of interest on an AlphaFold predicted structure of the pIII-C monomer. The disulfide bond stabilizing the hairpin in pIII-C is colored in *yellow*. Using ChimeraX, the pLDDT color scheme is represented from *blue* (bad) to *red* (good). *B*, bacterial two-hybrid assay in BTH101 reporter cells producing the indicated proteins or domains (pIII-C: residues 256–406; pIII-275: residues 275–406; pIII-286: residues 286–406; and pIII-308: residues 308–406 of pIII) fused to the T18 or T25 domain of the *Bordetella* adenylate cyclase were spotted on plates supplemented with IPTG X-Gal. Interaction between the two fusion proteins is attested by the *blue color*. The PgsA–PgsA interaction serves as the positive control. ICS, infection competence segment.

In order to define the segment of pIII responsible for TolQ and TolR binding, truncated versions of pIII-T18 were constructed (Figs. 5*A* and S1) based on the protein secondary structures (21). Our results showed that the last 132 residues of pIII-C (segment 275–406 of the mature protein) are sufficient for interaction with TolQ and TolR (Fig. 5*B*). TolR is a membrane protein anchored by its TolR1 domain and exposing a large domain in the periplasm (TolR2–3; residues 45–143). We wondered if pIII and TolR were interacting *via* their periplasmic domains. However, we found that the soluble pIII-N1, pIII-N2, and pIII-C_ΔTM_ constructs are not able to interact with TolR2–3 in a BACTH experiment (Fig. 2*B*).

Periplasmic production of the soluble pIII-N1 domain (48) or full-length pIII protein production in *E. coli* cells (Fig. S3) results in increased resistance to detergent deoxycholate (DOC) and to colicin A, as well as a 4-log decrease in the sensitivity of cells to phage uptake. This has been previously suggested to result from TolA sequestration by the phage protein, an interaction involving the C-terminal domain of TolA and the pIII-N1 YGT motif (48). In accordance with this hypothesis, we observed that cells producing the pIII_ΔYGT_ protein variant showed a level of sensitivity to DOC and to colicin A similar to the WT control cells. However, cells producing pIII_ΔYGT_ were less susceptible to fd phage infection (2- to 3-log decrease) compared with the control. Thus, pIII_ΔYGT_ still competes with phage uptake although less efficiently than the WT pIII protein. We questioned if this competition was occurring through the C-terminal domain of pIII. Production of pIII-C from a pBAD expression vector in WT cells did not result in *tol* phenotypes as the cells showed normal resistance to DOC, a WT level of phage infection and of sensitivity to colicin A despite correct expression and membrane localization of the protein (Fig. S3, *A*–*D*). As the pIII-C domain interacts with TolQ and TolR (Fig. 5), these data suggest that these interactions do not prevent the normal functioning of the Tol system in the cell or alternatively that the isolated pIII-C domain cannot interfere with a preassembled TolQRA complex.

### *In vivo* disulfide bond formation identifies interfaces between pIII-C, TolR, and TolQ TMHs

The cysteine-scanning approach has been proven efficient for studying *in vivo* the assembly and dynamic of the TolQ–TolR–TolA TMHs in the IM (25, 29). To gain further insight into pIII multimerization and interaction with TolQ and TolR, we introduced single cysteine mutations in the pBAD-pIII-C vector. We reasoned that thiol groups of cysteine side chains would form disulfide bonds if located at distances of 7 Å or less at the interface between interacting proteins. The selected positions (F381C, A382C, and F383C) encompass a full α-helix turn at the beginning of the pIII-C membrane anchor, close to the periplasmic side of the IM. Importantly, the native pIII-C sequence also possesses two cysteine residues (C354 and C371) predicted to be engaged in an intramolecular SS bond and strictly required for pIII insertion into the phage particle. Whole living cells producing pIII-C WT or cysteine variants were treated with the oxidative agent copper (II) orthophenanthroline (CuOP) to promote disulfide bond formation and analyzed using nonreducing SDS-PAGE conditions. Immunodetection indicated that the pIII-C variants (mature protein theoretical molecular weight [MW]: 16.2 kDa) accumulated at similar levels in *E. coli* cells. The 381C, 382C, and 383C substitutions promoted pIII-C homodimerization in the presence of CuOP, whereas the native cysteine C354 and C371 (WT condition) did not (Fig. S4).

**Figure 6:**
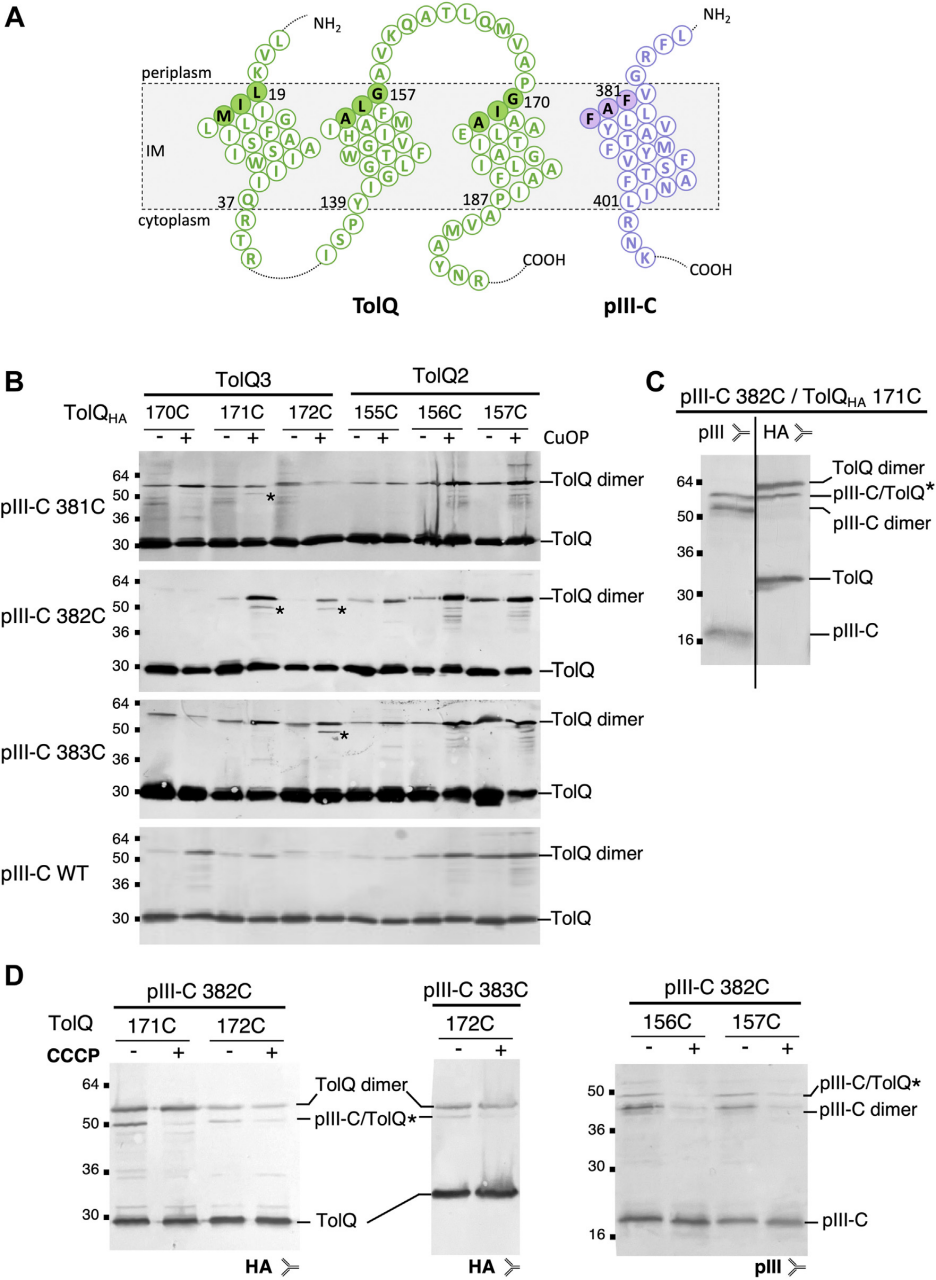
**pIII-C anchor interacts with TolQ TMH2 and TMH3 in a PMF-dependent fashion.***A*, schematic representation of the TMHs of TolQ and pIII-C, highlighting the residues analyzed in the cysteine scanning assay. *B*, cells producing the indicated pIII-C cysteine substitution in combination with the TolQ_HA_ cysteine mutations were treated or not with the oxidative agent copper (II) orthophenanthroline (CuOP) to increase dimer formation, then boiled in Laemmli buffer in the absence of a reducing agent, loaded onto 12.5% acrylamide SDS-PAGE, and immunodetected with anti-HA antibody. The positions of TolQ and TolQ dimer are indicated on the *right*. The signal susceptible to phage infection corresponds to the pIII-C–TolQ heterodimer and is indicated by a *star*. *C*, the pIII-C 382C/TolQ_HA_ 171C sample was reanalyzed by cutting the nitrocellulose membrane in two, and the signal was immunodetected by either the anti-HA antibodies (*right part of the panel*) or the anti-pIII antibody (*left part of the panel*). The molecular weight markers are indicated on the *left*. *D*, the experiments were conducted with (+) or without (−) addition of the protonophore CCCP before the CuOP treatment. The molecular weight markers (kilodalton) are indicated on the *left*. CCCP, carbonyl cyanide m-chlorophenylhydrazone; HA, hemagglutinin; PMF, proton motive force; TMH, transmembrane helix.

We then defined more precisely the organization of the pIII–TolQ and pIII–TolR interaction interfaces based on the intermolecular cysteine cross-linking data (Fig. 6*A*). Cysteine mutants in the first helix turn at the periplasmic side of the TolQ1 (19C, 20C, and 21C), TolQ2 (155C, 156C, and 157C), TolQ3 (170C, 171C, and 172C), and TolR1 (34C, 35C, and 36C) TMHs have been previously described (25, 29). Analysis of some of the TolQ_HA_ cysteine mutants (theoretical MW = 30 kDa) under CuOP condition reveals a TolQ dimer (60 kDa) as well as an additional signal of apparent MW ∼50 kDa (Fig. 6*B*). For combinations involving pIII-C 381C with TolQ 171C, pIII-C 382C with TolQ 171C and TolQ 172C, and finally pIII-C 383C with TolQ 172C, the 50 kDa signal was clearly seen by immunodetection using anti-HA (Fig. 6*B*) and anti-pIII antibodies (Fig. S5*A*). The TolQ_HA_ 171C/pIII-C 382C sample was reanalyzed on a separated Western blot, the nitrocellulose membrane was cut in half vertically, and the 50 kDa band was recognized by both the anti-HA and anti-pIII antibodies (Fig. 6*C*), further demonstrating that the complex includes both pIII and TolQ. In Fig. S5*A*, heterocomplexes between pIII-C and TolQ2 TMH were observed using anti-pIII antibodies (pIII-C 382C with TolQ 156C and TolQ 157C) (Fig. S5*A*) but not with anti-HA antibodies because of multiple product degradation signals (Fig. 6*B*). Of note, we occasionally observed that TolQ 170C formed weak heterocomplexes with pIII-C. Interestingly, no intermolecular disulfide bond could be formed between TolQ1 and pIII-C THMs (Fig. S5*B*).

**Figure 7:**
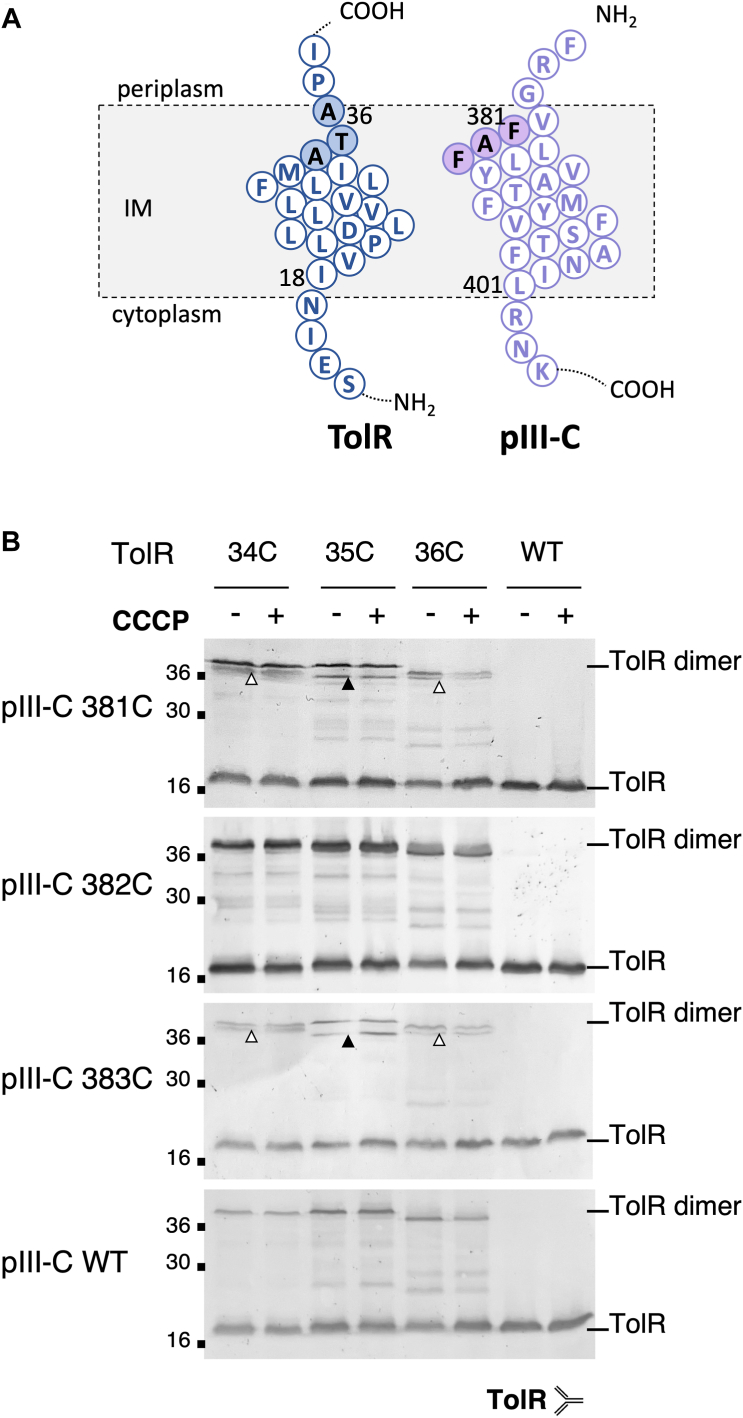
**pIII-C homodimerization and heterodimerization with TolR.***A*, schematic representation of the TMHs of TolR and pIII-C, highlighting the residues analyzed in the cysteine scanning assay. *B*, cells producing the indicated pIII-C cysteine substitution in combination with the TolR cysteine mutations were treated with CuOP, then boiled in Laemmli buffer in the absence of reducing agent, loaded onto 12.5% acrylamide SDS-PAGE, and immunodetected by the anti-TolR polyclonal antibody. The positions of TolR and TolR dimer are indicated on the *right*. The signal susceptible to phage infection corresponds to the pIII-C–TolR heterodimer and is indicated by a *triangle* (*black*: strong signal; *white*: faint signal). The experiments were conducted with (+) or without (−) addition of the protonophore CCCP before the CuOP treatment. The molecular weight markers (in kilodalton) are indicated on the *left*. CCCP, carbonyl cyanide m-chlorophenylhydrazone; CuOP, copper (II) orthophenanthroline; TMH, transmembrane helix.

**Figure 8:**
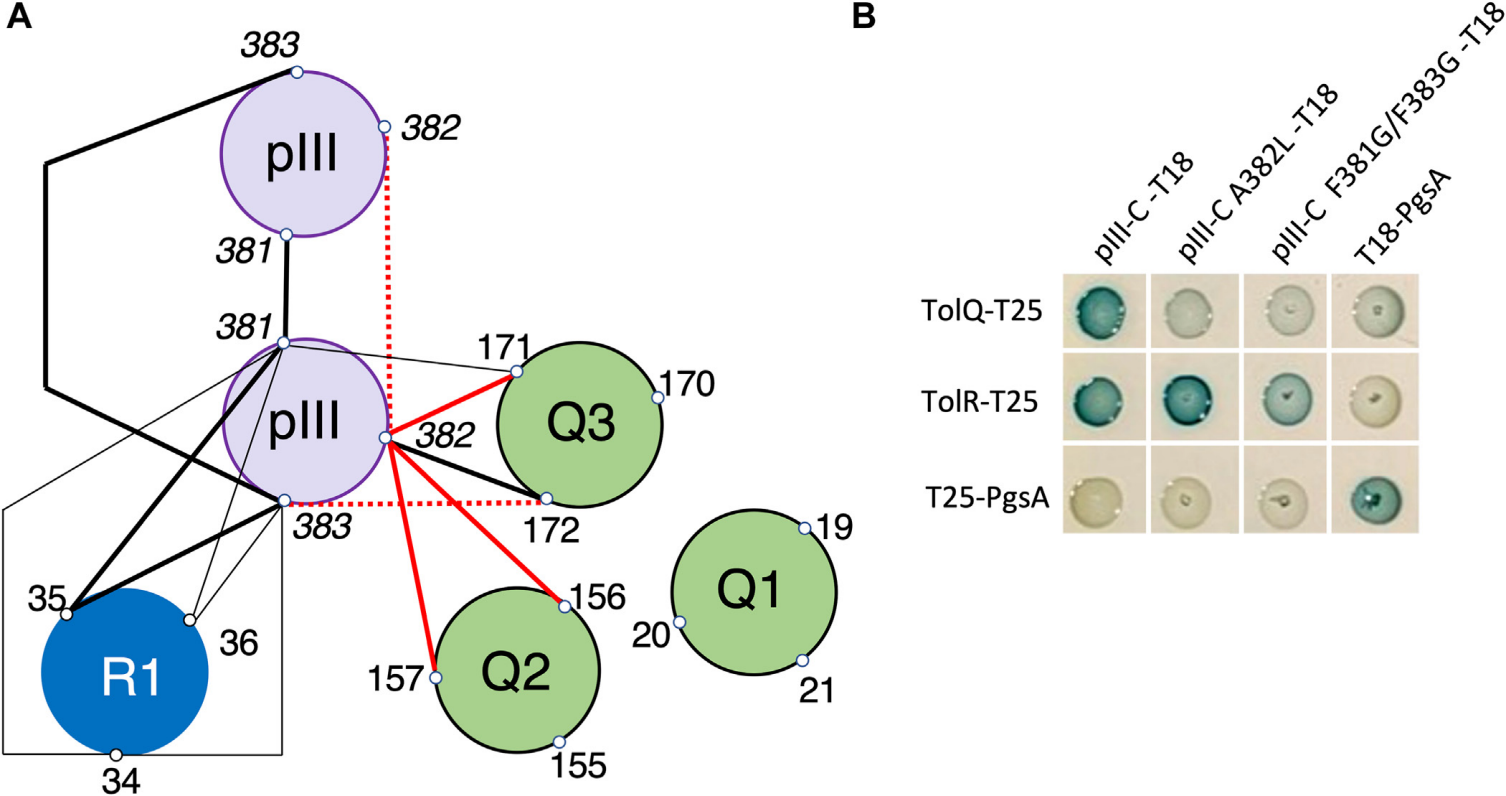
**Mapping of the interaction interfaces between pIIIC, TolQ, and tolR**. *A*, summary of the interactions observed by cysteine scanning. The TolR anchor (R1), the TolQ transmembrane helices (Q1, Q2, and Q3), and the C-terminal hydrophobic helix of pIII are represented as *circles* viewed from the periplasm. Residues were numbered on the outside of the *circles* and located at the periplasmic side of the TMHs and were mutated to cysteines. *Solid lines* indicate strong and reproducible cysteine crosslinking between indicated positions, *dashed lines* indicate faint interaction signals, and *red lines* are heterocomplexes lost upon treatment with the protonophore CCCP. *B*, bacterial two-hybrid assay in the BTH101 reporter cells producing the indicated proteins. The experiment was conducted as described in Figure 2. CCCP, carbonyl cyanide m-chlorophenylhydrazone.

We performed a similar screening between pIII-C and TolR (Fig. 7, *A* and *B*). Each individual TolR cysteine mutant was able to dimerize in CuOP oxidative conditions at an apparent MW of 36 kDa. pIII-C and TolR have very close MWs (16.2 kDa and 15.5 kDa, respectively) and tend to migrate similarly in the SDS-PAGE, which makes it difficult to discriminate homodimers from heterodimers, with the exception of the TolR 35C. A pIII-C–TolR heterocomplex was clearly detected for TolR 35C coproduced with pIII-C 381C and 383C. A faint heterodimer signal was also observed in some of our experiments with TolR 34C and 36C as well as pIII-C 381C and 383C. However, pIII-C 382C did not react with any of the tested positions of TolR in our assay. Overall, these data provide an initial mapping of the positioning of pIII-C, TolQ, and TolR THM relative to each other in the IM, which is summarized in Figure 8*A*.

In order to test the importance of the interaction interfaces identified, an A382L single mutation and a F381G–F383G double mutation were introduced into the pIII-C-T18 protein. We observed that both mutants lost their ability to interact with TolQ-T25, but not with T25-TolR, in a BACTH assay (Fig. 8*B*). Finally, we constructed virions carrying the point mutations A382L or F381G–F383G of the pIII protein. Titer determination by spectrophotometry showed that both mutant phages were produced, albeit at a slightly lower concentration than the WT fd-Tc (2.5-fold decrease). Virion stability testing indicated that both mutant phages were sensitive to sarkosyl detergent, whereas WT phages were only disassembled in the presence of SDS, demonstrating that residues F381, A382, and F383 are important for the overall stability of the phage head (Fig. S6*C*). Interestingly, fd-Tc phages pIII_F381G–F383G_ are no longer infectious, whereas fd-Tc virions pIII_A382L_ can still infect *E. coli*, although infection efficiency is reduced by about three logs compared with WT fd-Tc phages (Fig. S6*B*). Thus, the stability of virions in detergents is not strictly related to their infection efficiency.

### Dissipation of the PMF affects the infection process, independently of the reception step

The energy requirement for phage uptake is still an intriguing question, as it is challenging to differentiate between the energy consumed for the reception step (ATP-dependent F-pilus assembly and retraction), the translocation step of the phage in the periplasm, and the final transport of the phage DNA in the cytoplasm, coupled with ATP-dependent replication. In a previous study, we identified that TolQ T145A and T178A mutants unable to support PMF-dependent functions of the Tol-pal system were still infected by the phage, whereas the TolR D23A mutant was resistant (14). We decided to test the role of the PMF independently of the pilus reception step by studying phage infection in F^−^ cells. Indeed, cells lacking F-pilus, such as the W3110 strain, can be infected by filamentous phages when treated with CaCl_2_ (16). Carbonyl cyanide m-chlorophenylhydrazone (CCCP) can bind and transport protons across the cell IM, making it a powerful uncoupler even for short and low-concentration exposure (35). W3110 F^−^ cells were preincubated with 10 μM CCCP for 3 min to dissipate the PMF; then, the protonophore was removed before phage addition in 50 mM CaCl_2_ buffer. After incubation, cells were subject to two rounds of vortex and washes to remove free phages and reversibly adherent phages. Then, cells were incubated in LB for 10 min for metabolism recovery before enumeration of the infected cells on tetracycline-supplemented LB plates. In this assay, we observed that the CCCP-treated cells were 200-fold less susceptible to phage infection than the control experiment, whereas the treatment had no impact on cell survival (Table S1).

### The PMF status of the cell influences pIII heterodimerization with TolQ but not with TolR

As the TolQ–TolR motor is powered by the PMF, we wondered the interaction interfaces between pIII, TolQ, and TolR TMHs were linked to the energy status of the system. For the cysteine positions that displayed the most reproducible TolR/pIII-C and TolQ/pIII-C heterodimer signals, the cells were treated with the protonophore CCCP prior to the CuOP treatment. Interactions were monitored by *in vivo* disulfide bond formation and immunodetection (Figs. 6*D* and 7). Abolition of the cell PMF had no effect on the formation of the pIII-C–TolR heterocomplexes in the IM (Fig. 7). However, we observed that the formation of some pIII-C–TolQ heterodimers was strongly affected and even abolished in the absence of PMF (pIII-C 382C/TolQ 171C; pIII-C 382C/TolQ 156C; pIII-C 382C/TolQ 157C), whereas others were moderately affected or unaffected (pIII-C 382C/TolQ 172C; pIII-C 383C/TolQ 172C) (Fig. 6*D*). Interestingly, we occasionally observed (about 50% of our blots) that homodimerization of pIII-C 382C was also dependent on the PMF, whereas that of pIII-C 381C and pIII-C 383C were not (Fig. 6*D*, *right panel*). Altogether, these data suggest that the pIII TMH responds to the PMF to adopt a specific position relative to TolQ but not to TolR (Fig. 8). The pIII TMH (VFAFLLYVATFMYVFSTFANIL) is mainly composed of hydrophobic residues, with the exception of the polar residues T389, S395, T396, and N399 that could be relevant for functional or structural roles. Indeed, in the TolQ–TolR complex, highly conserved threonine residues within the TolQ2 (T145) and TolQ3 (T178) TMHs are proposed to stabilize the protonation of TolR D23, similarly to what is observed in the homologous ExbB–ExbD and MotA–MotB motors (27, 49, 50). To investigate this hypothesis, we compared the fd pIII-C TM sequence to other filamentous phage adhesion proteins that have been reported to bind to TolA in the literature, namely the coliphages If1 and IKe and the vibriophage CTX. Alignment of their C-terminal TM helices highlights a global conservation of the polar positions for the coliphages but not the vibriophage (Fig. S6*A*). We constructed fd phages carrying single or double mutations at these positions of interest (T389A–T396A; S395A; N399A) and determined that the phage variants produced were as infectious as the WT virus (Fig. S6*B*). Thus, none of the polar residues of the C-terminal hydrophobic helix of pIII is strictly required for successful infection.

## Discussion

Filamentous phages are often mistakenly considered as simplistic viruses, as their genome is “simply” covered by a helical assembly of major coat proteins forming a flexible capsid, which ends with a couple of minor coat proteins at both extremities. However, these particles are highly resistant to harsh environments and do not disassemble easily. For the past 10 years, the mechanistic model of phage translocation across the bacterial periplasm during infection has evolved little, conserving black boxes in the process. Bennet *et al.* (21) previously identified that the unlocking of the fd virion in the final step of infection is mediated by the C domain of pIII. More precisely, the C2 α-helix in pIII-C is essential for both infection and phage stability, as it folds over the hydrophobic pVI protein in the phage head and thus isolates it from the hydrophilic environment. This helix is comprised of the 28-long sequence required for efficient infection and previously referred to as the infection competence segment (Fig. 5*A*). It has been suggested that the phage head transitions between a closed and stable state and an open state allowing the C-terminal hydrophobic helices of pVI and pIII to insert into the membrane. This opening would require the active displacement of the C2 α-helix, possibly through a twist at the L4 linker (12). However, this hypothesis does not explain why phages deleted from the C2 domain, and thus in an “open” state, are less infectious than the WT phages (12). In this study, we formally demonstrate that pIII directly interacts with both the TolQ and TolR proteins *in vivo* by BACTH and co-IP assays. A truncated pIII-C domain comprising residues 275 to 406 of the protein is required for interaction with TolQ and TolR in a BACTH assay. Conversely, shorter versions of pIII-C (residues 286–406), and therefore lacking the entire C2 helix, cannot interact with TolQ (Fig. 5). These results suggest that C2 might be directly or indirectly involved in pIII-C interaction with TolQ (but not TolR) and provide an explanation for its requirement for efficient infection (12). Finally, we observed that pIII protects the approximate 80 terminal residues of TolQ from proteolysis, which correspond to the periplasmic loop and the TMH3 of TolQ (Fig. S2*B*). As proteinase K digests proteins preferentially after hydrophobic or aromatic residues, we believe that TolQ Ala162 or Leu164 might be masked by pIII binding and that the periplasmic portion of TolQ might bind to pIII. AlphaFold modelization of the pIII-C–TolQ complex indeed position the L164 and A162 residues at the interface between the two partners, the pIII ß-hairpin loop burying part of the TolQ loop in the structure (Fig. S7, *A* and *B*). Interestingly, the L164 residue is one of the most conserved positions in the TolQ and ExbB periplasmic loops (Fig. S2*B*). Overall, TolQ and ExbB share 65.3% identity and 77.5% similarity on the TMH2–loop–THM3 sequence, which could explain why ExbB can interact with pIII-C (Fig. S2*A*) and supports phage import in the absence of TolQR (18). Furthermore, the conservation of the TolQ TMH2–loop–TMH3 sequence and TolR TMH in *V. cholerae* (100% identity on the transmembrane domains, 83.33% identity on the loop segment; Fig. S2*B*) and of TolR THM may also explain why a hybrid coliphage composed of the CTXΦ pIII N1–N2 domains fused to the FfΦ pIII-C domain is infectious to *V. cholerae* (51).

This pattern is reminiscent of the translocation determinants identified for several colicins, which are bacterial toxins that rely on a subset of the Tol proteins for uptake (52). For example, colicin N, colicin A, and colicin K are pore-forming toxins that require TolA, TolQ, and TolR (and TolB for ColA and ColK) for translocation. Colicin K was found to bind to TolA, TolQ, and TolR independently (53).

To determine how pIII, TolR, and TolQ interact in the IM, we performed a cysteine scanning of pIII and provided a first mapping of the organization of the TMHs relative to each other. First, the interaction signals between pIII and TolQ THM define a heterodimerization interface involving Q2 (156, 157) and Q3 (171, 172) but not Q1. Interestingly, in the TolQ–TolR motor, Q2, Q3, and TolR TMHs delimit the channel by which the ions transit. Q2 and Q3 are oriented toward the center of the complex and the dimer of TolR, whereas Q1 is at the periphery where it interacts with the transmembrane anchor of TolA (31, 32). We show here that the pIII–TolQ and the pIII–TolR interactions can be observed independently. One important question that remains to be addressed is to determine if pIII-C interacts simultaneously or sequentially with TolQ and TolR. To date, complex structure predictions using AlphaFold does not fully fit our cysteine cross-linking data (Fig. S7, *C*–*E*). This *in silico* approach did not allow us to model a complex including the three proteins, since pIII-C was excluded from the TolQ–TolR heterodimer in the prediction (Fig. S7, *F* and *G*).

The other intriguing question is the role played by TolQ and TolR when the phage reaches the IM. A possibility is that contact with the TolQR complex triggers the extraction of the pIII C-terminal helix and its positioning into the IM. TolQ–TolR form the proton-dependent motor required for TolA mechanical movement in the periplasm. According to our data, pIII-C does not position itself at the periphery of the motor against Q1, as is the case with TolA. Thus, our work supports the idea that pIII does not mimic TolA to harvest the energy from the TolQ–TolR complex to trigger phage head opening. These findings are also consistent with our previous work demonstrating that strains producing assembled but nonfunctional motors (TolQ T145A and TolQ T178A variants) are still susceptible to phage infection (18). In contrast, the identified interactions suggest that the phage protein pIII mobilizes TolQ and TolR TMHs in a specific arrangement distinct from the TolQRA 5:2:1 complex. The interaction between the phage and TolA could be the first step in the process, transmitting long-distance structural rearrangements in TolQR, in pIII-C, or in both partners. One possibility is that pIII-C induces disassembly of the TolQ–TolR motor complex or the displacement of one or several units of TolQ or TolR from the TolQR complex. This would trigger the extraction of the hydrophobic helix of pIII-C from the particle and its burial in the IM. Alternatively, pIII-C could mobilize free TolQ and TolR proteins not engaged into the motor complexes. However, the fact that infection relies on the existence of assembled Q–R–A complexes (18) does not support the latter hypothesis. There is currently no information on the nature of the pore allowing the phage to transfer its DNA through the IM, although it probably comprises a pentamer of pIII and possibly of pVI. The TMH of TolQ and/or TolR might also be part of the pore. To date, it is challenging to reconcile the results and faces of TM segments in interaction in the TolQR complex. The fact that pIII-C homodimers and heterodimers with TolR do not point out a defined interface suggests that movements of helices in the IM may occur, as it is the case for TolR homodimers (29). However, we observed that residues A382 and F381/F383 are important for pIII–TolQ interaction (Fig. 8*B*) and probably for the structuring of the phage head (Fig. S6*C*), these data being consistent with their position in the virion structure recently solved (Protein Data Bank [PDB] ID: 8B3O) (12). The mechanism by which pIII–TolQ interaction responds to the PMF (Fig. 6*D*) remains to be determined. The recent breakthrough in the experimental resolution of the homologous ExbB–ExbD complex structure by cryo-EM may now serve the study of pIII-C interaction with TolQR (26).

In conclusion, our studies suggest a novel host-assisted step in the molecular process used by filamentous phages for gaining entry into bacteria. The adhesion protein pIII confirms here its role as a pilot involved in each step of the translocation process, from the extracellular environment to the cell IM, through its organization in distinct specialized subdomains. These findings will help to understand the structural basis for filamentous phage Tol-mediated membrane penetration mechanisms in a broad range of biomedically important bacterial pathogens.

## Experimental procedures

### Bacterial strains, medium, and growth conditions

Bacterial strains and plasmids used in this study are listed in Table S2. Bacteria were routinely cultivated in LB at 37 °C with agitation at 160 rpm. When indicated, antibiotics were added to the medium at the following concentrations: ampicillin (50 or 100 μg/ml), kanamycin (50 μg/ml), and tetracycline (15 μg/ml).

### Plasmid construction

Sequence amplifications by PCRs were performed using Q5 High Fidelity DNA polymerase (NEB) and pairs of primers (Sigma–Aldrich) listed in Table S2. Plasmids were constructed using standard cloning techniques, as previously described (18, 25). Point mutants on expression plasmids were obtained by Quick-change site-directed mutagenesis using complementary pairs of oligonucleotides (Table S2) and Pfu Turbo polymerase (Agilent). All constructs were confirmed by DNA sequencing (Eurofins, MWG).

### Protein–protein interaction assays

#### BACTH assay in *E. coli* BTH101 and Oxi-Blue strains

The Oxi-Blue strain used for two-hybrid experiments is a *lac+* derivative of the Oxi-BTH strain previously engineered to study interactions between proteins with disulfide bonds (46). Briefly, the Oxi-BTH strain (*lacZ*^*−*^) is deleted for glutaredoxin reductase (*gor*) and thioredoxin reductase (*trxB*) genes, which allows disulfide bond formation in the cytoplasm, whereas cytoplasmic expression of the isomerase DsbC promotes correct disulfide bond formation for proteins containing more than two cysteines. The *lacZ* gene was transduced in this background using a P1-lysate of an *E. coli* K12 lacZ+ strain (Gift from Dr E. Bouveret). The resulting strain, named Oxi-Blue, can be screened on either X-Gal or MacConkey plates. Interaction experiments by the bacterial adenylate cyclase–based two-hybrid technique (BACTH) were conducted as previously published (46). The experiments were done at least in triplicate, and a representative assay is shown.

### Protein modeling

AlphaFold2 and AlphaFold multimer webserver interfaces (54) were used to model the protein structures. The available structures of pIII-N1 (PDB ID: 1TOL) and pIII-N1-N2 (PDB ID: 1G3P) were retrieved from the Protein Data Base. Visual representations of the structures were prepared with ChimeraX (UCSF ChimeraX) (55).

### *In vivo* disulfide bond formation and immunodetection

Cysteine scanning was carried out as previously described (29) with slight modifications. About 8 × 10^8^ exponentially growing W3110 cells were harvested and resuspended in 1 ml of 20 mM sodium phosphate buffer (PBS; pH 6.8) and then treated for 10 min with 2.5 mM *N*-ethylmaleimide (Sigma) to block reduced thiol groups. When required, cells were treated with the oxidative catalyst 0.3 mM CuOP (Sigma) for 15 min prior washing in sodium phosphate buffer and blocking with *N*-ethylmaleimide for 20 min. After centrifugation, cell pellets were resuspended in Laemmli loading buffer in the absence of the reducing agent. For disulfide bond formation experiments in energy depletion conditions, cells were first treated with 40 μM CCCP (Sigma) for 15 min prior to CuOP labeling.

### Co-IP

Co-IP experiments were performed as previously described (53). Exponentially growing cells (2 × 10^9^) were collected, washed with 20 ml of 10 mM sodium phosphate buffer (NaPi [pH 6.8]), and resuspended in NaPi buffer supplemented with 1% formaldehyde. Cells were incubated at room temperature for 20 min, the cross-linking reaction was stopped by the addition of 0.3 M Tris–HCl (pH 6.8), and the cells were finally washed in 20 mM Tris–HCl (pH 6.8). The membrane proteins were solubilized for 30 min at 37 °C in TES (10 mM Tris–HCl, pH 7.5, 5 mM EDTA, 1% SDS) in the presence of protease inhibitors (cOmplete; Roche) and diluted 15-fold in TNE (10 mM Tris–HCl, pH 7.5, 150 mM NaCl, 5 mM EDTA) supplemented with 1% Triton X-100. After incubation for 2 h at room temperature with vigorous shaking, the extract was centrifuged for 30 min at 14,000*g* to remove unsolubilized material. One milliliter of each supernatants was then incubated overnight at 4 °C with anti-HA agarose beads (Cell Signaling Technology; catalog no.: 3956S) or Protein-A sepharose CL-4B beads (GE Healthcare) customized with 2 μl antibodies against pIII (Mobitech). Beads were then washed twice with TNE supplemented with 1% Triton X-100, once in TNE supplemented with 0.1% Triton X-100 and 0.1% Tween, and once in TNE supplemented with 0.1% Triton X-100. The immunoprecipitated material was heated in loading buffer prior to analyses by SDS-PAGE and immunoblotting.

### SDS-PAGE and immunoblotting

Protein samples resuspended in 2× loading buffer (100 mM Tris–HCl [pH 6.8], 2% SDS, 10% glycerol, 0.01% bromophenol blue, and 5% 2-β-mercaptoethanol) were subjected to SDS-PAGE. For detection by immunostaining, proteins were transferred onto nitrocellulose membranes, and immunoblots were probed with primary antibodies followed by goat secondary antibodies coupled to alkaline phosphatase and developed in alkaline buffer in the presence of 5-bromo-4-chloro-3-indolylphosphate and nitroblue tetrazolium. The anti-TolA3 and anti-TolR polyclonal antibodies are from our laboratory collection, whereas the anti-pIII monoclonal antibody (NEB), the anti-HA monoclonal antibody (Invitrogen), and alkaline phosphatase–conjugated goat anti-rabbit and antimouse antibodies (Millipore) have been purchased as indicated.

## Data availability

All data for this work are contained in this article.

## Supporting information

This article contains supporting information (18, 25, 29, 46, 56, 57, 58, 59).

## Conflict of interest

The authors declare that they have no conflicts of interest with the contents of this article.
