# Supplementary material for: Direct interaction between fd phage pilot protein pIII and the TolQ–TolR proton-dependent motor provides new insights into the import of filamentous phages
Source: J Biol Chem. 2023 Jul 13;299(8):105048. doi: 10.1016/j.jbc.2023.105048 (PMC10424213; doi:10.1016/j.jbc.2023.105048)
Supplement: Supporting information [file mmc1.pdf]

Direct interaction between fd phage pilot protein pIII and the TolQ-TolR proton-dependent motor provides new insights into the import of filamentous phages.

Callypso PELLEGRI, Ambre MOREAU, Denis DUCHE and Laetitia HOUOT

Supporting information

Content:

Supporting Figures and Tables

Supporting data: Experimental procedures

Supporting bibliography

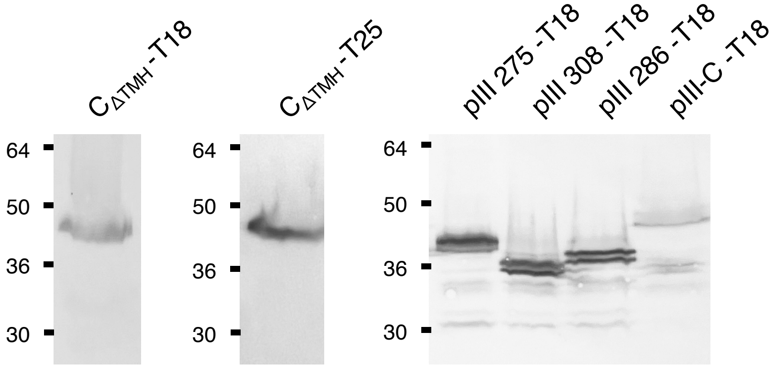

Fig. S1. Protein production level of various BACTH constructs used in this study. Western immunoblot of 0.2 OD units of whole-cell lysates of *E. coli* carrying various inducible expression vectors, and probed with monoclonal anti-Cya 3D1 (T18 constructs) or anti-pIII (NEB, T25 constructs) antibodies. The molecular weight markers (in kDa) are indicated on the left.

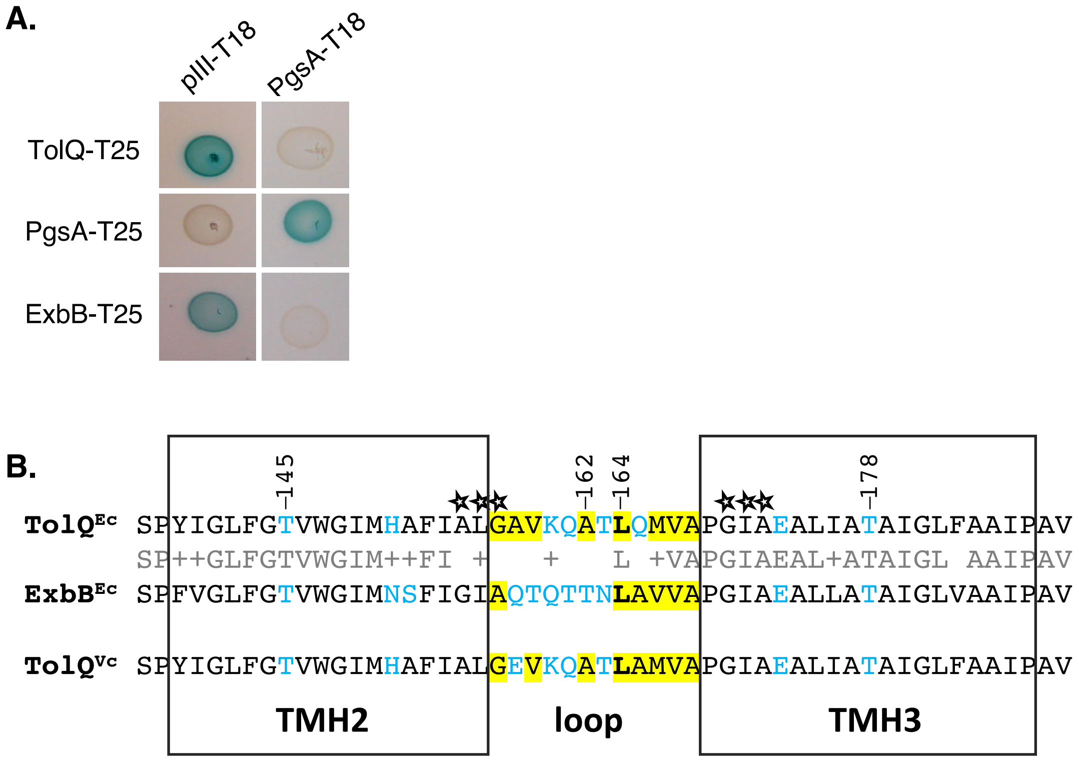

Fig.S2. Cross-talk between TolQ and ExbB for phage uptake. *A*) Bacterial two-hybrid assay in BTH101 reporter cells producing the indicated proteins fused to the T18 or T25 domain of the *Bordetella* adenylate cyclase and spotted on plates supplemented with IPTG X-Gal. PsgA homodimerization serves as a positive control. *B*) Sequence alignment of TolQ and ExbB from *E. coli* or *V. cholerae* and restricted to the periplasmic loop surrounded by the second and third trans-membrane helices (TMH, boxed). Polar residues (blue), hydrophobic residues in the periplasmic loop (yellow), positions mutated in the cysteine-scanning experiment (stars).

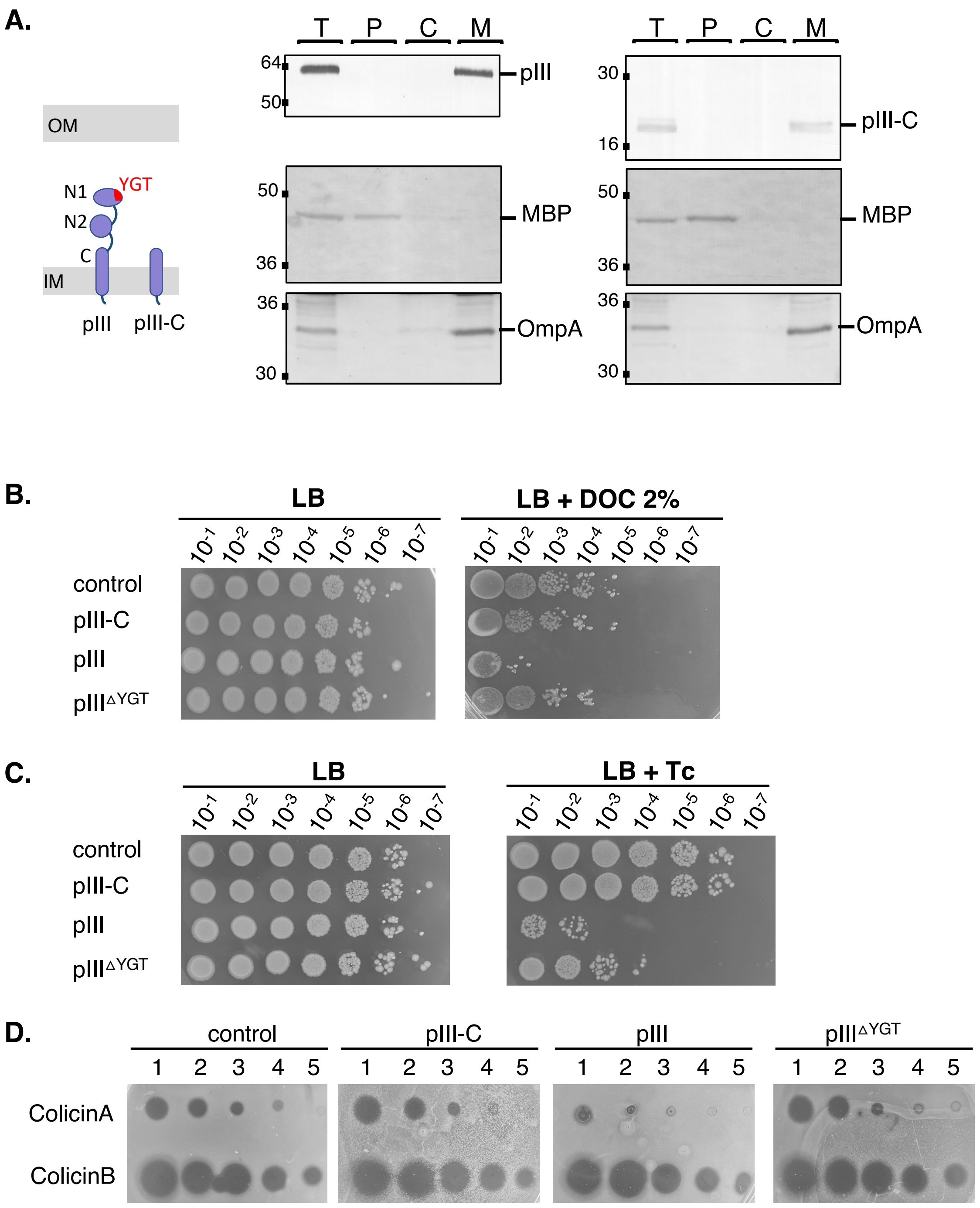

Fig.S3. Phenotypic characterization of GM1 strains producing phage pIII, pIII$^{\Delta YGT}$ or pIII-C proteins. *A*) Fractionation assay attesting the localization of the produced proteins. Left panel is a schematic representation of the protein in the cell envelope. The YGT motif previously identified as essential for pIII-N1/TolA3 interaction is indicated. Central and right panels : the fractions were loaded on a 12.5% acrylamide SDS-PAGE and immunodetected using anti-pIII, anti-MBP and anti-OmpA antibodies. T: total fraction, P: periplasmic, C: cytoplasmic, M: membrane. The molecular weight markers (in kDa) are indicated on the left. B) Cell growth on LB and LB supplemented with deoxycholate 2%. C) Susceptibility to phage infection. Cells were incubated with the phage during 30 min, 10-fold serial diluted and spotted on LB plates and on LB plates supplemented with tetracycline (LB+Tc, right panel), in order to numerate the total number of CFU, and fd-Tc phage infected CFU, respectively. Experiments were conducted in triplicate. D) Colicin sensitivity was estimated by 5-fold serial spot dilutions. One microliter of colicin (A or B) was spotted on to a growing lawn of cells. Clear zones indicate cell death. ColA is dependent on the Tol system while ColB is dependent on the TonB-ExbBD system for uptake.

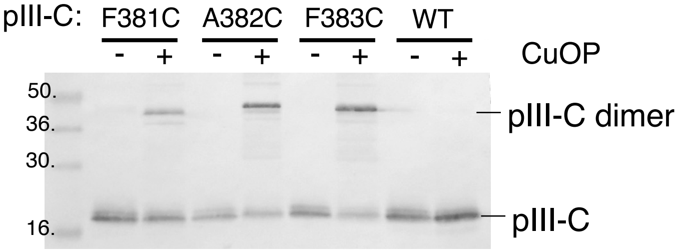

Fig. S4: Dimerization of pIII-C visualized by in vivo disulfide bond formation .
Cells producing the indicated pIII-C cysteine substitution were treated or not with the oxidative agent copper (II) orthophenanthroline to increase dimer formation, then boiled in Laemmli buffer in absence of reducing agent, loaded onto 12.5% acrylamide SDS-PAGE and immunodetected with anti-pIII antibody. The molecular weight markers (in kDa) are indicated on the left.

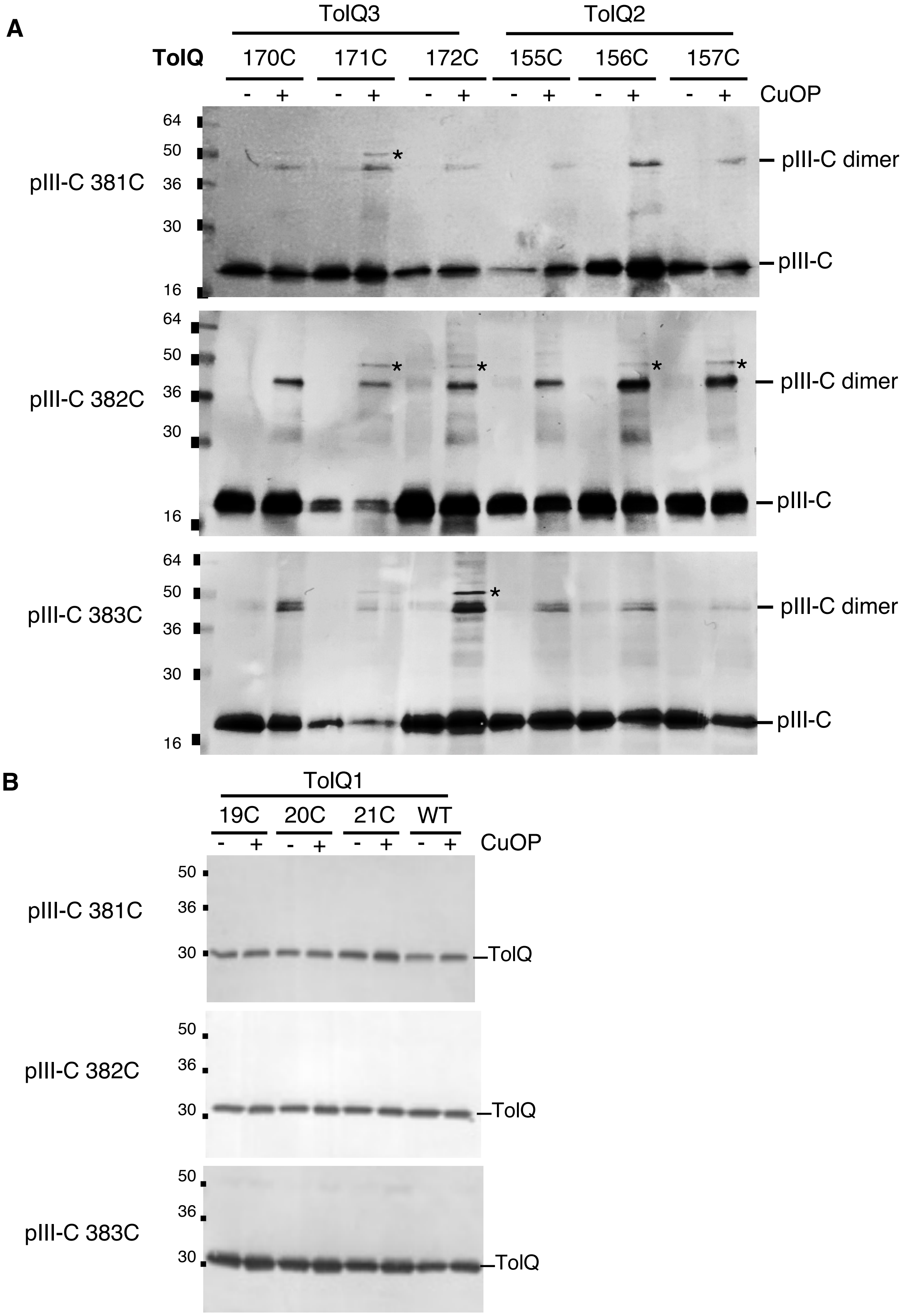

Fig. S5: pIII-C interacts with TolQ TMH2 and TMH3 but not TMH1.

Cells producing the indicated pIII-C cysteine substitution in combination with the $TolQ_{HA}$ cysteine mutations in TolQ2 and TolQ3 (A) or TolQ1 (B) were treated or not with the oxidative agent copper (II) orthophenanthroline (CuOP) to increase dimer formation, then boiled in Laemmli buffer in absence of reducing agent, loaded onto 12.5% acrylamide SDS-PAGE and immunodetected with the anti-pIII (A) or anti HA antibody (B). The positions of pIII-C and pIII-C dimer are indicated on the right. The signal susceptible to correspond to the pIII-C/TolQ heterodimer is indicated by a star. The molecular weight markers (in kDa) are indicated on the left.

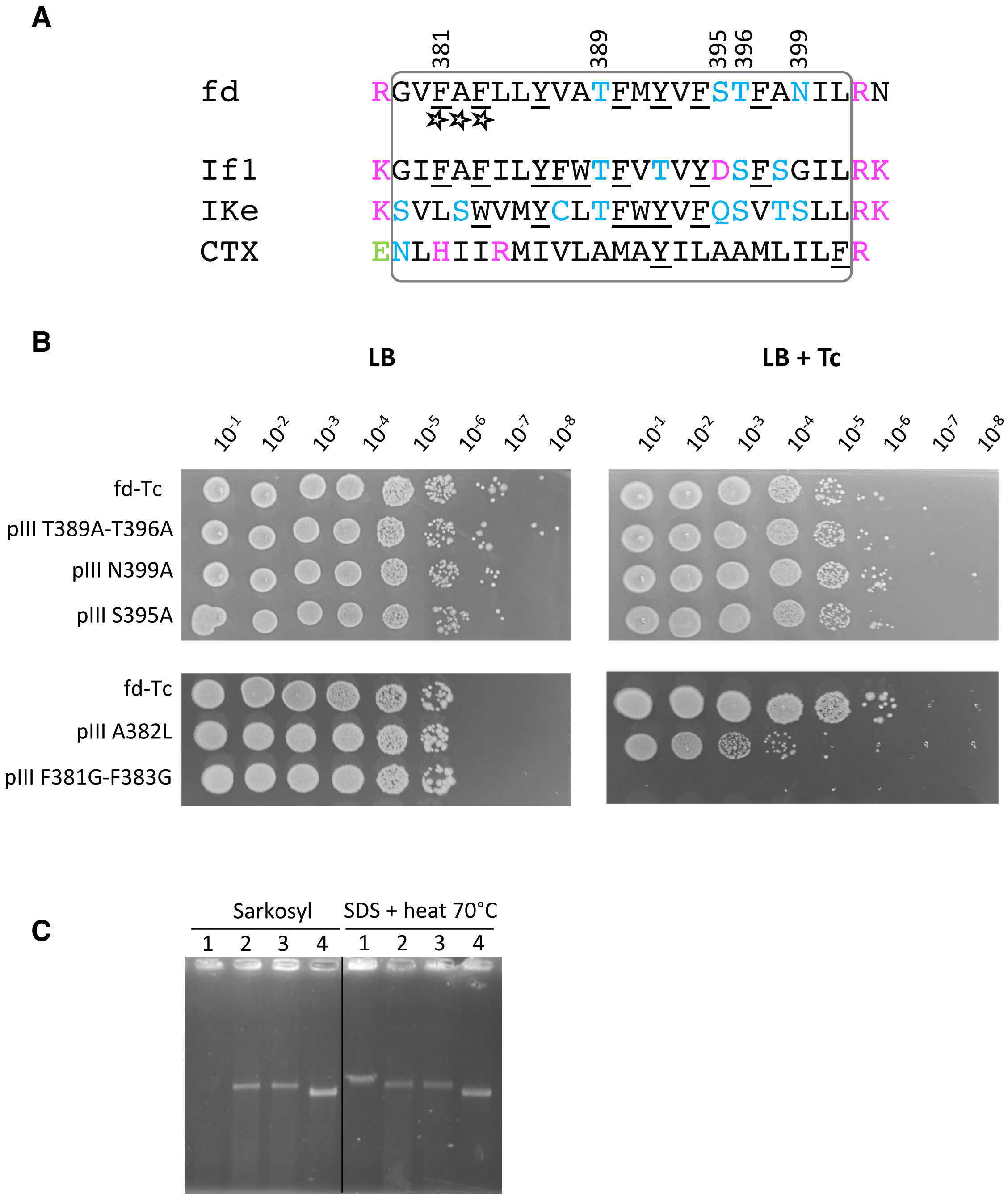

Fig. S6: Analysis of pIII C-terminal membrane anchor. A. Sequence alignment of filamentous phage predicted TMHs. The hydrophobic helix of the coliphages fd, If1, Ike and the vibriophage CTX are presented boxed. Polar residues (blue), aromatic residues (underlined), acidic residues (green), basic residues (pink), positions mutated in the cysteine-scanning experiments (Fig. 6 and 7) and in the phage infection assay (stars).
B. Host susceptibility to phage mutants. GM1 F^{+} cells were incubated with the WT fd-Tc phages or the indicated virion mutants during 30 min, 10-fold serial diluted and spotted on LB plates and on LB plates supplemented with tetracycline (LB+Tc, right panel), in order to numerate the total number of CFU, and of fd-Tc phage infected CFU, respectively. Experiments were conducted in triplicate.
C. Phage virions stability assay by native agarose gel electrophoresis. Virions particles were produced with wild-type pIII (1), pIII A382L (2), pIII F381G-F383G (3) or pIII-286 (4). Virions were incubated either with sarkosyl 0.1% for 10 min (left panel) or with SDS 1% and heated at 70°C for 15 min (right panel) prior to electrophoresis on a 0.6% agarose gel. Released ssDNA from destabilized virions can be visualized by gelred staining. The phage sample pIII-286 used as a control was previously shown to be unstable and to release its genome in the presence of sarkosyl detergent (Rakonjac et al. 1999).

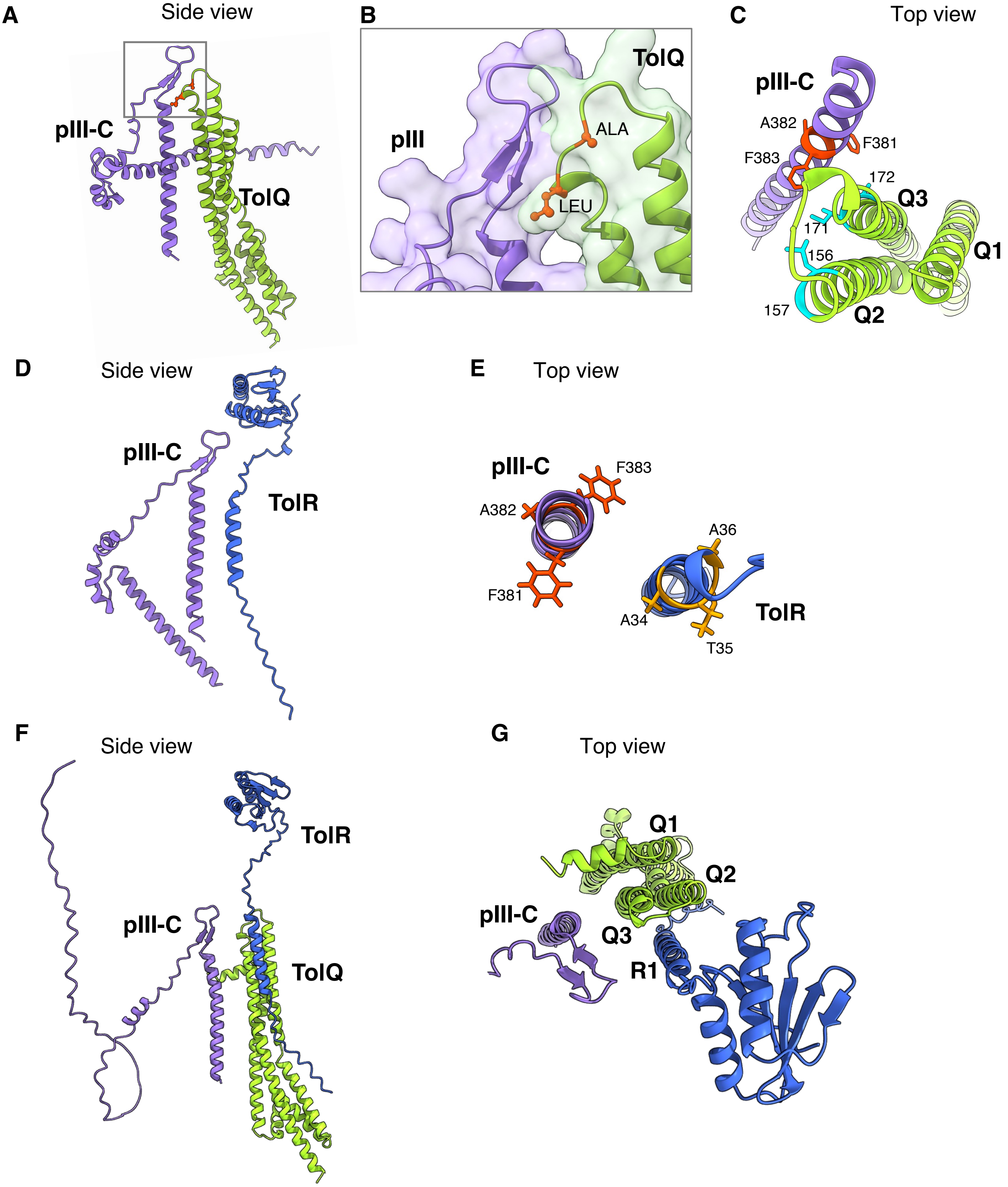

Fig. S7: Modelization of pIII interaction with TolQ and TolR.

Alphafold predictions of the pIII-C domain (coloured in purple), TolQ (yellow-green) and TolR (blue). In the predicted pIII-C/TolQ complex, pIII-C terminal TMH is positioned obliquely to TolQ TMH2 and TMH3, with the β-hairpin hiding part of the TolQ periplasmic loop. The boxed region is detailed in panel B, with the TolQ residues Ala162 and Leu164 colored in red. The pIII-C/TolR complex prediction is presented in panel D. Panel C and E are top views of the complexes from the periplasm. Residues mutated in the cysteine crosslinking experiment are indicated (C and E). The prediction comprising pIII-C, TolQ and TolR (panel F and G) identify the TolQ/TolR heterocomplex, but excludes pIII-C from the structure.

		Total CFU	SD	Infected CFU/mL	SD	F	SD
W3110	CCCP -	1.33 x 10E+10	6.11 x 10E+09	7.85 x 10E+03	2.17 x 10E+03	6.21 x 10E-07	1.08 x 10E-07
W3111	CCCP +	1.60 x 10E+10	6.93 x 10E+09	4.7 x 10E+01	2.31 x 10E+01	2.83 x 10E-09	2.89 x 10E-10

Table S1. Effect of the CCCP on the infection frequency for the W3110 F$^-$ strain . Cells pretreated or not with the protonophore CCCP 10 µM were incubated with the fd-Tc phage in the presence of $CaCl_2$ during 15 min, 10-fold serial diluted and spotted on LB plates to numerate total CFU, and LB plates supplemented with tetracycline to numerate the fd-Tc infected CFU, respectively. Experiments were conducted in triplicate. The frequency of infection (F) was calculated as the mean of the 3 infection with standard deviation (SD).

Table S2: Strains, plasmids and oligonucleotides

Strain, plasmid or library	Genotype or description	Reference or source
***E. coli* strains and phages**		
DH5α	F^- Φ80*lac*ZΔM15 Δ(*lac*ZYA-*arg*F) U169 *rec*A1 *end*A1 *hsd*R17 (rK–, mK+) *pho*A *sup*E44 λ– *thi*-1 *gyr*A96 *rel*A1	Laboratory collection
W3110	F- lambda- IN(rrnD-rrnE)1 rph-1	Laboratory collection
W3110 Δ*tolQR*Δ*exbBD*	W3110 strain deleted of *tolQ*, *tolR* and *exbB, exbD* genes	Samire *et al.*, 2020
GM1	*ara, thi,Δ(lac pro),* F', *lac, pro*	Laboratory collection
BTH101	F-, cya-99, araD139, galE15, galK16, rpsL1 (StrR), hsdR2, mcrA1, mcrB1.	Laboratory collection
Oxi-Blue	Shuffle T7 Express (*fhuA2 lacZ::T7 gene1* [lon] *ompT ahpC gal λatt*::pNEB3-r1-*cDsbC* ($Spec^R$, $lacI^q$) *ΔtrxB* sulA11 *R(mcr-73::miniTn10*--Tet^S)2 [dcm] *R(zgb-210::Tn10* --Tet^S) *endA1 Δgor Δ(mcrC-mrr)114::IS10), Δcya, ΔphoA* $lacZ^+$	Pellegri *et al.,* unpublished
fd-Tc	fd phage carrying a tetracyclin resistance gene	ATCC 37000
fd-Tc pIII T389A T396A	fd-Tc bearing a T-to-A substitution at position 389 and 395 in pIII	This study
fd-Tc pIII N399A	fd-Tc bearing a N-to-A substitution at position 369 in pIII	This study
fd-Tc pIII S395A	fd-Tc bearing a S-to-A substitution at position 399 in pIII	This study
fd-Tc pIIIΔN2	fd-Tc deleted of the pIII-N2 domain (residues 72 to 256).	This study
fd-Tc pIII A382L	fd-Tc bearing a A-to-L substitution at position 382 in pIII	This study
fd-Tc pIII F381G/F383G	fd-Tc bearing a F-to-G substitution at position 381 and 383 in pIII	This study
fd-Tc pIII-286	fd-Tc bearing a deletion of residues 1 to 285 in pIII. The virion is unstable in the presence of sarkosyl 0.1%.	This study, based on Rakonjac *et al*. 1999
**Plasmids used**		
pOK12	IPTG-inducible plasmid, KanR	Vieira and Messing, 1991
pBAD/HisC	pBR322-derived expression vector, L-arabinose inducible, AmpR	Invitrogen
pKT25	BACTH expression vector encoding T25 fragment of *B. pertussis cyaA*; Km^R	Karimova *et al*., 1998
pUT18	BACTH expression vector encoding T18 fragment of *B. pertussis cyaA*; Amp^R	Karimova *et al*., 1998
pUT18C	Modified version of pUT18 with the polylinker located on the C-terminal end of T18	Karimova *et al*., 1998
Two-hybrid constructions		
pIII-T18	fd pIII sequence cloned upstream T18 into pUT18	This study
pIIIC-T18	fd pIII sequence (residues 256 to 406) cloned upstream T18 into pUT18	This study
pVI-T18	fd pIII sequence cloned upstream T18 into pUT18C	This study
N1-T18	fd pIII sequence (residues 1 to 71) cloned upstream T18 into pUT18C	This study
N2-T18	fd pIII sequence (residues 82 to 222) cloned upstream T18 into pUT18	This study
$C_{\Delta TM}$-T18	fd pIII sequence (residues 256 to 378) cloned upstream T18 into pUT18	This study
pIII275-T18	fd pIII sequence (residues 275 to 406) cloned upstream T18 into pUT18	This study
pIII286-T18	fd pIII sequence (residues 286 to 406) cloned upstream T18 into pUT18	This study
pIII308-T18	fd pIII sequence (residues 308 to 406) cloned upstream T18 into pUT18	This study
pIIIC-T18 A382L	pIIIC-T18 plasmid bearing a A-to-L substitution at position 382 in pIII	This study
pIIIC-T18 F383W	pIIIC-T18 plasmid bearing a F-to-W substitution at position 383 in pIII	This study
pIIIC-T18 F381G F383G	pIIIC-T18 plasmid bearing a F-to-G substitution at position 381 and 383 in pIII	This study
T18-TolR2-3	*E.coli* TolR sequence (residues 45 to 143) cloned downstream T18 into pUT18	Battesti and Bouveret. 2008
PgsA-T18	*E.coli* PgsA sequence cloned upstream T18 into pUT18	Battesti and Bouveret. 2008
pIII-T25	fd pIII sequence cloned upstream T25 into pKT25	This study
pIIIC-T25	fd pIII sequence (residues 256 to 406) cloned upstream T25 into pKT25	This study
N1-T25	fd pIII sequence (residues 1 to 71) cloned upstream T25 into pKT25	Houot *et al.,* 2017
N2-T25	fd pIII sequence (residues 82 to 222) cloned upstream T25 into pKT25	This study
$C_{\Delta TM}$-T25	fd pIII sequence (residues 256 to 378) cloned upstream T25 into pKT25	This study
TolQ-T25	*E.coli* TolQ sequence cloned upstream T25 into pKT25	This study
T25-TolR	*E.coli* TolR sequence cloned downstream T25 into pKT25	This study
T25-TolR2-3	*E.coli* TolR sequence (residues 45 to 143) cloned downstream T25 into pKT25	Battesti and Bouveret. 2008
ExbB-T25	*E.coli* ExbB sequence cloned upstream T25 into pKT25	This study
PgsA-T25	*E.coli* PgsA sequence cloned upstream T25 into pKT25	Battesti and Bouveret. 2008
Co-IP, physiology and infection assay constructions		
pOK-$TolQ^{HA}$	pOK12 plasmid carrying the *E. coli tolQ* gene fused to an HA tag, Kan^R	Zhang *et al*., 2011
pOK-TolR	pOK12 plasmid carrying the *E. coli tolR* gene, Kan^R	Zhang *et al*., 2009
pBAD-pIII	pBAD/HisC plasmid carrying the fd *pIII* gene, Amp^R	This study
pBAD-pIIIC	pBAD/HisC plasmid carrying the fd *pIII* gene fragment coding residues 275 to 406, Amp^R	This study
pBAD-$pIII^{\Delta YGT}$	Deletion of the sequence encoding the pIII YGT motif (residues 54 to 56) in the pBAD-pIII plasmid, AmpR	This study
Cystein scanning constructions		
pBAD-pIIIC-F381C	pBAD-pIIIC plasmid bearing a F-to-C substitution at position 381 in pIIIC, Amp^R	This study
pBAD-pIIIC-A382C	pBAD-pIIIC plasmid bearing a A-to-C substitution at position 382 in pIIIC, Amp^R	This study
pBAD-pIIIC-F383C	pBAD-pIIIC plasmid bearing a F-to-C substitution at position 383 in pIIIC, Amp^R	This study
pOK-$TolQ^{HA}$-L19C	pOK-$TolQ^{HA}$ plasmid bearing a L-to-C substitution at position 19 in TolQ, Kan^R	Zhang *et al*., 2011

pOK-TolQ^{HA}-I20C	pOK-TolQ^{HA} plasmid bearing a I-to-C substitution at position 20 in TolQ, Kan^{R}	Zhang *et al*., 2011
pOK-TolQ^{HA}-M21C	pOK-TolQ^{HA} plasmid bearing a M-to-C substitution at position 21 in TolQ, Kan^{R}	Zhang *et al*., 2011
pOK-TolQ^{HA}-A155C	pOK-TolQ^{HA} plasmid bearing a A-to-C substitution at position 155 in TolQ, Kan^{R}	Zhang *et al*., 2011
pOK-TolQ^{HA}-L156C	pOK-TolQ^{HA} plasmid bearing a L-to-C substitution at position 156 in TolQ, Kan^{R}	Zhang *et al*., 2011
pOK-TolQ^{HA}-G157C	pOK-TolQ^{HA} plasmid bearing a G-to-C substitution at position 157 in TolQ, Kan^{R}	Zhang *et al*., 2011
pOK-TolQ^{HA}-G170C	pOK-TolQ^{HA} plasmid bearing a G-to-C substitution at position 170 in TolQ, Kan^{R}	Zhang *et al*., 2011
pOK-TolQ^{HA}-I171C	pOK-TolQ^{HA} plasmid bearing a I-to-C substitution at position 171 in TolQ, Kan^{R}	Zhang *et al*., 2011
pOK-TolQ^{HA}-A172C	pOK-TolQ^{HA} plasmid bearing a A-to-C substitution at position 172 in TolQ, Kan^{R}	Zhang *et al*., 2011
pOK-TolR-A34C	pOK-TolR plasmid bearing a A-to-C substitution at position 34 in TolR, Kan^{R}	Zhang *et al*., 2009
pOK-TolR-T35C	pOK-TolR plasmid bearing a T-to-C substitution at position 35 in TolR, Kan^{R}	Zhang *et al*., 2009
pOK-TolR-A36C	pOK-TolR plasmid bearing a A-to-C substitution at position 36 in TolR, Kan^{R}	Zhang *et al*., 2009

**Oligonucleotide**	**Name**	**Sequence (5' → 3')**
pIII-T18	oLH456<br>oLH457	gcggataacaatttcacacaggaaacagctATGAAAAAATTATTATTCGCAATTCC<br>GGCGGCTGAATTCGAGCTCGGTACCgcAGACTCCTTATTACGCAGTATGTTAGC
pIIIC-T18	oLH489<br>oLH490	AGTTGTTCCTTTCTATTCTCACTCCGCTTCCGGTGATTTTGATTATGAAAAAATGGC<br>GCCATTTTTTCATAATCAAAATCACCGGAAGCGGAGTGAGAATAGAAAGGAACAACT
pVI-T18	oLH468<br>oLH469	caatttcacacaggaaacagctATGACCATGCCAGTTCTTTTGGGTATTCCG<br>GCTCGGTACCCGGGGATCCTCTAGAGTTTTATCCCAATCCAAATAAGAAACG
N1s-T18	oLH517<br>oLH518	GAACGCCACTGCAGGTCGACTCTAGCTGAAACTGTTGAAAGTTGTTTAGC<br>GAATTCGAGCTCGGTACCCGGGGATCCTGAGCCACCACCCTCATTTTCAGG
N2s-T18	oLH519<br>oLH520	CTGCAGGTCGACTCTAGAGGATCCCTCTGAGGGTGGCGGTACTAAACCTC<br>CCCGTGGCCTCGCTGGCGGCTGAATTCGAACCAGAGCCGCCGCCAGCATTGA
Cs-T18	oLH515<br>oLH516	cagctATGACCATGATTACGCCAAGCGATTTTGATTATGAAAAAATGGC<br>CCTCTAGAGTCGACCTGCAGGCATGCAAACGGAATAAGTTTATTTTGTCAC
pIII132-T18	oLH511<br>oLH512	CCTTTAGTTGTTCCTTTCTATTCTCACTCCgcaGCCGATGAAAACGCGCTACAGTCTG<br>GAATAGAAAGGAACAACTAAAGGAATTGCG
pIII121-T18	oLH491<br>oLH492	AGTTGTTCCTTTCTATTCTCACTCCGCTGGCAAACTTGATTCTGTCGCTACTGATTACG<br>CGTAATCAGTAGCGACAGAATCAAGTTTGCCAGCGGAGTGAGAATAGAAAGGAACAACT
pIII93-T18	oLH493<br>oLH494	AGTTGTTCCTTTCTATTCTCACTCCGCTGGCCTTGCTAATGGTAATGGTGCTACTGG<br>CCAGTAGCACCATTACCATTAGCAAGGCCAGCGGAGTGAGAATAGAAAGGAACAACT
pIII-T25	oLH502<br>oLH503	CCAGCCTGATGCGATTGCTGCATGGTtgctcctgctccACGGAATAAGTTTATTTTGTCAC<br>GTTTGCGTAACCAGCCTGATGCGATTGCTGtcctcctcctgctgctgcAGACTCCTTATTACGCAGTATGTTAGC
pIIIC-T25	oLH489<br>oLH490	AGTTGTTCCTTTCTATTCTCACTCCGCTTCCGGTGATTTTGATTATGAAAAAATGGC<br>GCCATTTTTTCATAATCAAAATCACCGGAAGCGGAGTGAGAATAGAAAGGAACAACT
N2s-T25	oLH521<br>oLH522	CTGCAGGGTCGACTCTAGAGGATCCCtctgagggtggcggtactaaacctc<br>aaaacgacggccgaattcttagTTAcgaccagagccgccgccagcattgac
Cs-T25	oLH513<br>oLH514	ggataacaatttcacacaggaaacagctATGGATTTTGATTATGAAAAAATGGC<br>CCAGCCTGATGCGATTGCTGCATGGTtgctcctgctccACGGAATAAGTTTATTTTGTCAC
TolQ-T25	oLH504<br>oLH506	caatttcacacaggaaacagctATGGTGACTGACATGAATATCCTTG<br>CAGCCTGATGCGATTGCTGCATGGTCCCCTTGTTGCTCTCGCTAAC
TolR-T25	oLH204<br>oLH205	GTCGACTCTAGAGGATCCCCGGGTACCTAAGGCCAGAGCGCGTGGACGAGGT<br>gacgttgtaaaacgacggccgaattcttagTTAGATAGGCTGCGTCATTAAAC
ExbB-T25	oLH202<br>oLH203	GCGGATAACAATTTCACACAGGAAACAGCTGTGGGTAATAATTTAATGCAGACGG<br>ACCAGCCTGATGCGATTGCTGCATGGTCATTCCTGCGCGTAATTTTTGTGCGAC
pIIIC-T18 A382L	oLH596<br>oLH598	ATAAACTTATTCCGTGGTGTCTTTctGTTTCTTTTATATGTTGCCACC<br>AAAGACACCACGGAATAAGTTTATTTTGTCAC
pIIIC-T18 F381G<br>F383G	oLH602<br>oLH603	GTGACAAAATAAACTTATTCCGTGGTGTCggTGCGggtCTTTTATATGTTGCCACCTTTATGTATG<br>CCACGGAATAAGTTTATTTTGTCACAATCAATAGAAAATTC
pBAD-pIII	oLH474<br>oLH475	GGGCTAACAGGAGGAATTAACCATGGTGAAAAAATTATTATTCGCAATTCC<br>CTGTCCACCAGTCATGCTAGCCATACCttaAGACTCCTTATTACGCAGTATG
pBAD-pIIIC	oLH489<br>oLH490	AGTTGTTCCTTTCTATTCTCACTCCGCTTCCGGTGATTTTGATTATGAAAAAATGGC<br>GCCATTTTTTCATAATCAAAATCACCGGAAGCGGAGTGAGAATAGAAAGGAACAACT
pBAD-pIIIC-F381C	oLH383<br>oLH384	CTTATTCCGTGGTGTCTGTGCGTTTCTTTTATATG<br>ACGGAATAAGTTTATTTTGTCACAATC
pBAD-pIIIC-A382C	oLH395<br>oLH398	CCGTGGTGTCTTTtgcTTTCTTTTATATGTTGCC<br>AAAGACACCACGGAATAAGTTTATTTTGTCAC
pBAD-pIIIC-F383C	oLH396<br>oLH398	CCGTGGTGTCTTTGCGtgtCTTTTATATGTTGCC<br>AAAGACACCACGGAATAAGTTTATTTTGTCAC
fd-Tc pIII T389A T395A	oLH569<br>oLH570	GCAAACGCCGAAAATACATACATAAAGGCGGCAACATATAAAAGAAACGC<br>GCCGCCTTTATGTATGTATTTTCGGCGTTTGCTAACATACTGCGTAATAAGG
fd-Tc pIII N369A	oLH571<br>oLH572	CGACGTTTGCTgcCATACTGCGTAATAAGGAGTC<br>CGCAGTATGgcAGCAAACGTCGAAAATACATAC
fd-Tc pIII S399A	oLH472<br>oLH473	ATTTGCGACGTTTGCTAACATACTGCG<br>CGTCGCAAATACATACATAAAGGTGG
fd-Tc pIII A382L	oLH596<br>oLH598	ATAAACTTATTCCGTGGTGTCTTTctGTTTCTTTTATATGTTGCCACC<br>AAAGACACCACGGAATAAGTTTATTTTGTCAC
fd-Tc pIII F381G<br>F383G	oLH602<br>oLH603	GTGACAAAATAAACTTATTCCGTGGTGTCggTGCGggtCTTTTATATGTTGCCACCTTTATGTATG<br>CCACGGAATAAGTTTATTTTGTCACAATCAATAGAAAATTC
fd-Tc pIIIΔN2	oLH487<br>oLH488	GGCGGTTCTGAGGGTGGCGGTTCTGATGGCGGCTCCGGTTCCGGTGATTTTG<br>CAAAATCACCGGAACCGGAGCCGCCATCAGAACCGCCACCCTCAGAACCGCC
fd-Tc pIII-286	oLH491<br>oLH492	AGTTGTTCCTTTCTATTCTCACTCCGCTGGCAAACTTGATTCTGTCGCTACTGATTACG<br>CGTAATCAGTAGCGACAGAATCAAGTTTGCCAGCGGAGTGAGAATAGAAAGGAACAACT

Experimental procedures

Cell fractionation. Cell fractionation was performed as previously described (55). The membrane pellet was resuspended in 1 ml of 1 M carbonate sodium (Na_2CO_3) and incubated on a rotating wheel for 30 min at room temperature. Ultracentrifugation at 90,000 g for 40 min then separated the integral membrane fraction (pellet) from the membrane-associated fraction (supernatant). The periplasmic, cytoplasmic, and membrane-associated fractions were precipitated with 15% trichloroacetic acid and resuspended in loading buffer prior to analysis by SDS-PAGE and immunoblotting.

Sensitivity test to DOC. Strains carrying the plasmid of interest were cultivated in the presence of L-arabinose (0.02%) to induce protein expression until they reached OD_{600nm}=0.6. Normalized cultures were serial diluted and spotted onto LB plates supplemented or not with deoxycholate 2%. After overnight incubation at 37 °C, survival was reported as the highest dilution of strain able to form colonies.

Colicin susceptibility. Colicin activities were tested as described (18). Briefly, over-night cultures of the strains were spread on LB agar petri dishes supplemented with antibiotics. Plasmid expression was induced using L-arabinose (0.02%) and IPTG (100 µM). After drying, 1 µL of serial dilutions (10 fold) of colicin A and colicin B were spotted on the bacterial lawn. Plates were incubated at 37°C for 16 hrs.

fd-Tc phage preparation and titration. fd-Tc phages were produced from an infected *E. coli* GM1 strain as described otherwhere (18) with the following modifications. After 16 hrs of culture, the cells were pelleted by two rounds of centrifugation at 5,000 g for 20 min. Phages were isolated from the supernatant using the isoelectric precipitation method (Mourez *et al*, 2004). Briefly, the phage suspension was brought to pH=4.6 with HCl and centrifuged at 13,000 g for 20 min in order to precipitate the particles. The phage pellet was rinsed with deionized water, centrifuged again at 13,000g for 10 min, and the phages were finally resuspended in PBS buffer 1X pH 7.0 and filter-sterilized (0.45 µm syringe filter). Phage preparations were checked for sterility by plating on LB plate. Titration of the WT phage suspension was performed using standard protocols (18). To calculate the number of mutant phage particles in each samples, absorbance readings were taken at 269 nm and 320 nm using a Carry-UV spectrophotometer (Agilent). The titer was calculated using the following formula (Abs. 269 - Abs. 320) × 6 × 1016/plasmid size, with 9183 nt for the fd-Tc genome (Mourez *et al*, 2004).

Susceptibility to fd-Tc phage infection assays. Strains of interest were cultivated to reach OD600nm =0.7 to 0.8 and normalized to the same initial OD600nm. For F+ infection, 10 µl of phage suspension was added to 200 µL GM1 F+ cells (multiplicity of infection of 1,000 phages per bacteria). Infection assay were performed in triplicate in 96-well plates, during 30 min of incubation at room temperature without shaking. The cells were vigorously homogenized by pipetting, and immediately serial diluted 10 fold in sterile PBS. 5 µl were drops on a LB plate (total recipient CFU) or LB agar supplemented with Tc (15 ng/µl) (phage infected CFU).
For F-independent infection, 450 µL of W3110 cells were treated with 10 µM CCCP for 3 min at room temperature. Cells were centrifuged 5 min at 8,000 rpm and the pellet was resuspended in 50 µL CaCl2 50 mM and incubated for 15min with 25 µL of phage suspension. The cells were submitted to vigorous vortexing and two rounds of centrifugation and wash in 700 µL sterile PBS in order to remove unattached and reversibly attached phages. Finally, the pellet was resuspended in 60 µL LB and incubated for 10 min at 37°C for recovery before serial dilution and spreading on LB or LB+Tc plates. After overnight incubation at 37°C, isolated CFU were counted in the appropriate dilution test. The frequency of infection (F) was determined by dividing the number of infected cells by the number of total recipient cells. Experiments were conducted in triplicates.

Phage stability assay by agarose gel electrophoresis. Virions samples were fully disassembled by incubation in 1% SDS-supplemented DNA loading dye (1X Tris–acetate–EDTA (TAE) buffer, 5% glycerol, 0.25% bromophenol blue pH 8.3) at 70 °C for 15 min. For stability assays, virions were mixed with 0.1% Sarkosyl-supplemented loading dye and incubated at room temperature for 10 min prior to electrophoresis. Samples were loaded onto TAE 1X agarose gels (0.6%). Electrophoresis were performed at 50V for two hours. The ssDNA released from the phage particles was visualized by staining the gel with Gelred dye for 45 min.

Supporting bibliography

Battesti, A., and Bouveret, E. (2008) Improvement of bacterial two-hybrid vectors for detection of fusion proteins and transfer to pBAD-tandem affinity purification, calmodulin binding peptide, or 6-histidine tag vectors. Proteomics. 8, 4768–4771

Houot, L., Navarro, R., Nouailler, M., Duché, D., Guerlesquin, F., and Lloubes, R. (2017) Electrostatic interactions between the CTX phage minor coat protein and the bacterial host receptor TolA drive the pathogenic conversion of Vibrio cholerae. Journal of Biological Chemistry. 292, 13584–13598

Karimova, G., Pidoux, J., Ullmann, A., and Ladant, D. (1998) A bacterial two-hybrid system based on a reconstituted signal transduction pathway. Proc. Natl. Acad. Sci. U.S.A. 95, 5752–5756

Mourez, M., and Collier, R. J. (2004) Use of Phage Display and Polyvalency to Design Inhibitors of Protein–Protein Interactions. in Protein-Protein Interactions, pp. 213–228, Humana Press, New Jersey, 261, 213–228

Vieira, J., and Messing, J. (1991) New pUC-derived cloning vectors with different selectable markers and DNA replication origins. Gene. 100, 189–194

Samire, P., Serrano, B., Duché, D., Lemarié, E., Lloubès, R., and Houot, L. (2020) Decoupling Filamentous Phage Uptake and Energy of the TolQRA Motor in Escherichia coli. J Bacteriol. 10.1128/JB.00428-19

Zhang, X. Y.-Z., Goemaere, E. L., Thomé, R., Gavioli, M., Cascales, E., and Lloubés, R. (2009) Mapping the Interactions between Escherichia coli Tol Subunits. Journal of Biological Chemistry. 284, 4275–4282

Zhang, X. Y.-Z., Goemaere, E. L., Seddiki, N., Célia, H., Gavioli, M., Cascales, E., and Lloubes, R. (2011) Mapping the Interactions between Escherichia coli TolQ Transmembrane Segments. Journal of Biological Chemistry. 286, 11756–11764
